# Subnanometric Cu clusters on atomically Fe-doped MoO_2_ for furfural upgrading to aviation biofuels

**DOI:** 10.1038/s41467-022-30345-0

**Published:** 2022-05-11

**Authors:** Xin Zhao, Fengliang Wang, Xiangpeng Kong, Ruiqi Fang, Yingwei Li

**Affiliations:** 1 State Key Laboratory of Pulp and Paper Engineering, School of Chemistry and Chemical Engineering, South China University of Technology, Guangzhou, 510640 China; 2 The School of Materials Science and Engineering, Harbin Institute of Technology, Shenzhen, 518055 China; 3 South China University of Technology−Zhuhai Institute of Modern Industrial Innovation, Zhuhai, 519175 China

**Keywords:** Biodiesel, Catalyst synthesis, Heterogeneous catalysis

## Abstract

Single cluster catalysts (SCCs) are considered as versatile boosters in heterogeneous catalysis due to their modifiable single cluster sites and supports. In this work, we report subnanometric Cu clusters dispersed on Fe-doped MoO_2_ support for biomass-derived furfural upgrading. Systematical characterizations suggest uniform Cu clusters (composing four Cu atoms in average) are homogeneously immobilized on the atomically Fe-doped ultrafine MoO_2_ nanocrystals (Cu_4_/Fe_0.3_Mo_0.7_O_2_@C). The atomic doping of Fe into MoO_2_ leads to significantly modified electronic structure and consequently charge redistribution inside the supported Cu clusters. The as-prepared Cu_4_/Fe_0.3_Mo_0.7_O_2_@C shows superior catalytic performance in the oxidative coupling of furfural with C_3_~C_10_ primary/secondary alcohols to produce C_8_~C_15_ aldehydes/ketones (aviation biofuel intermediates), outperforming the conventionally prepared counterparts. DFT calculations and control experiments are further carried out to interpret the structural and compositional merits of Cu_4_/Fe_0.3_Mo_0.7_O_2_@C in the oxidative coupling reaction, and elucidate the reaction pathway and related intermediates.

## Abstract (web summary)

Single cluster catalysts are considered as versatile boosters in heterogeneous catalysis. Here the authors report subnanometric Cu clusters dispersed on Fe-doped MoO_2_ support exhibits attractive performance in furfural valorization to C8-C15 aviation biofuels via a newly developed reaction route.

## Introduction

Downsizing metal active sites into several atoms scale is demonstrated effective in boosting their catalytic performances^1–3^. Compared with conventional metal nanoparticles/bulks, atomic site catalysts combine the advantages of both homogeneous and heterogeneous ones, such as extremely high atom utilization, well-defined active sites, and reliable durability, thus showing remarkably enhanced catalytic activities^4–6^. Consequently, single cluster catalysts (SCCs) are emerging, which compose of a certain number of metal atoms as active sites (M_x_, where M and x represent metal and atom number, respectively)^7,8^. Apart from the above-mentioned common merits of atomic site catalysts, the tunable composition and geometry of M_x_ may endow SCCs with other distinctive properties. Thus, a rational decoration of M_x_ could correspondingly modify its charge distributions and consequently affect the catalytic processes^9,10^.

Recently, numerous efforts have been devoted to the synthesis of novel SCCs with unique electronic structures. For instance, tailoring the metal type and atom number of M_x_ could effectively modify its geometrical topology. The distinctively spatial distribution of the metal atoms in M_x_ leads to unexpected size effect and polarized charge distribution, achieving remarkable catalytic performances in various reactions^11–14^. This protocol is highly dependent on the physicochemical natures of the metals employed (e.g., coordination number, chemical state, and geometry topology), which could only qualitatively change the electronic structure of SCCs. Alternatively, decorating the support of SCCs may modify the electronic structure of M_x_ in a more precise and selective way, i.e., altering the metal-support interactions^15–20^. In theory, the even introduction of different dopants into SCC supports could generally and uniformly modulate the electronic property of M_x_, which, however, is still scarcely investigated in spite of the significance in the pursuing of highly efficient single cluster catalysts for advanced catalytic applications.

In this work, subnanometric Cu clusters immobilized on Fe-doped MoO_2_ nanocrystals are fabricated via a cation exchange-diffusion strategy and subsequent pyrolysis procedure. The obtained nanocomposite features a multi-shelled hollow hierarchical porous octahedron (SHHPO) morphology. Spherical aberration-corrected high-angle annular dark-field scanning transmission electron microscopy (AC HAADF-STEM) and X-ray absorption spectroscopy (XAS) results uncover the homogeneous immobilization of Cu clusters composing four Cu atoms in average (Cu_4_) upon the atomically Fe-doped ultrafine MoO_2_ nanocrystals in the obtained Cu_4_/Fe_0.3_Mo_0.7_O_2_@C. The atomic doping of Fe into MoO_2_ leads to significantly modified electronic structure and consequently charge redistribution inside the Cu clusters. Moreover, the as-prepared Cu_4_/Fe_0.3_Mo_0.7_O_2_@C shows superior catalytic performance in the oxidative coupling of furfural (FFA) with C_3_~C_10_ primary/secondary alcohols (e.g., isopropanol) to produce the corresponding C_8_~C_15_ aldehydes/ketones (e.g., 4-(2-furyl)-buten-2-one, FBO) as aviation biofuel intermediates. The structural and compositional relationship of Cu_4_/Fe_0.3_Mo_0.7_O_2_@C in the oxidative coupling reaction and the possible reaction pathway are also investigated.

## Results

### Synthesis of Cu_4_/Fe_0.3_Mo_0.7_O_2_@C SHHPO

**Fig. 1:**
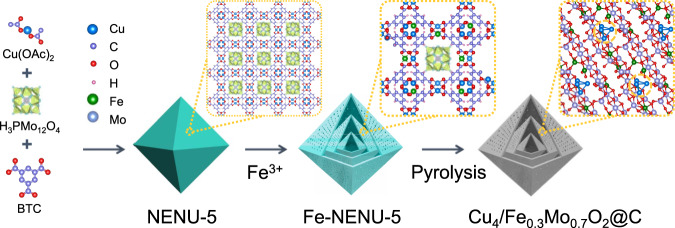
Schematic illustration of the synthesis procedure for Cu_4_/Fe_0.3_Mo_0.7_O_2_@C SHHPO. The synthesis involves the self-assembling of NENU-5, cation exchange-diffusion and pyrolysis procedures.

The synthesis of Cu_4_/Fe_0.3_Mo_0.7_O_2_@C SHHPO is illustrated in Fig. 1. First, NENU-5 is assembled by using a reported method^21^. Powder X-ray diffraction (XRD) patterns (Supplementary Fig. 1a) and scanning electron microscopy (SEM) images (Supplementary Fig. 1c–e) reveal the successful synthesis of monodispersed NENU-5 octahedrons in size of 1 µm with smooth external surfaces. N_2_ adsorption–desorption isotherms indicate the microporous nature of NENU-5 (Supplementary Fig. 2b, c). Subsequently, the obtained NENU-5 is immerged into a Fe^3+^-contained aqueous solution to substitute partial Cu ions with Fe species to yield Fe-NENU-5. Meanwhile, the Fe^3+^-contained aqueous solution could also facilitate the structural evolution of the solid NENU-5 octahedrons through cation exchanging to fabricate multi-shelled nanostructures. The XRD patterns of Fe-NENU-5 match well with those of the parent NENU-5 (Supplementary Fig. 2a), suggesting the well-retained crystalline phase of NENU-5 upon Fe^3+^ substitution. The as-prepared Fe-NENU-5 features typical type IV N_2_ adsorption–desorption isotherms with an obvious N_2_-uptake increment at high relative pressures (0.9–1.0) (Supplementary Fig. 2b), revealing the generation of meso- and macropores. Correspondingly, the formation of hierarchical pores in Fe-NENU-5 is also confirmed by the significantly reduced specific surface area (from 615.2 to 399.3 m^2^ g^−1^, Supplementary Table 1) and enlarged pore-size distribution range (Supplementary Fig. 2c) in comparison with NENU-5.

**Fig. 2:**
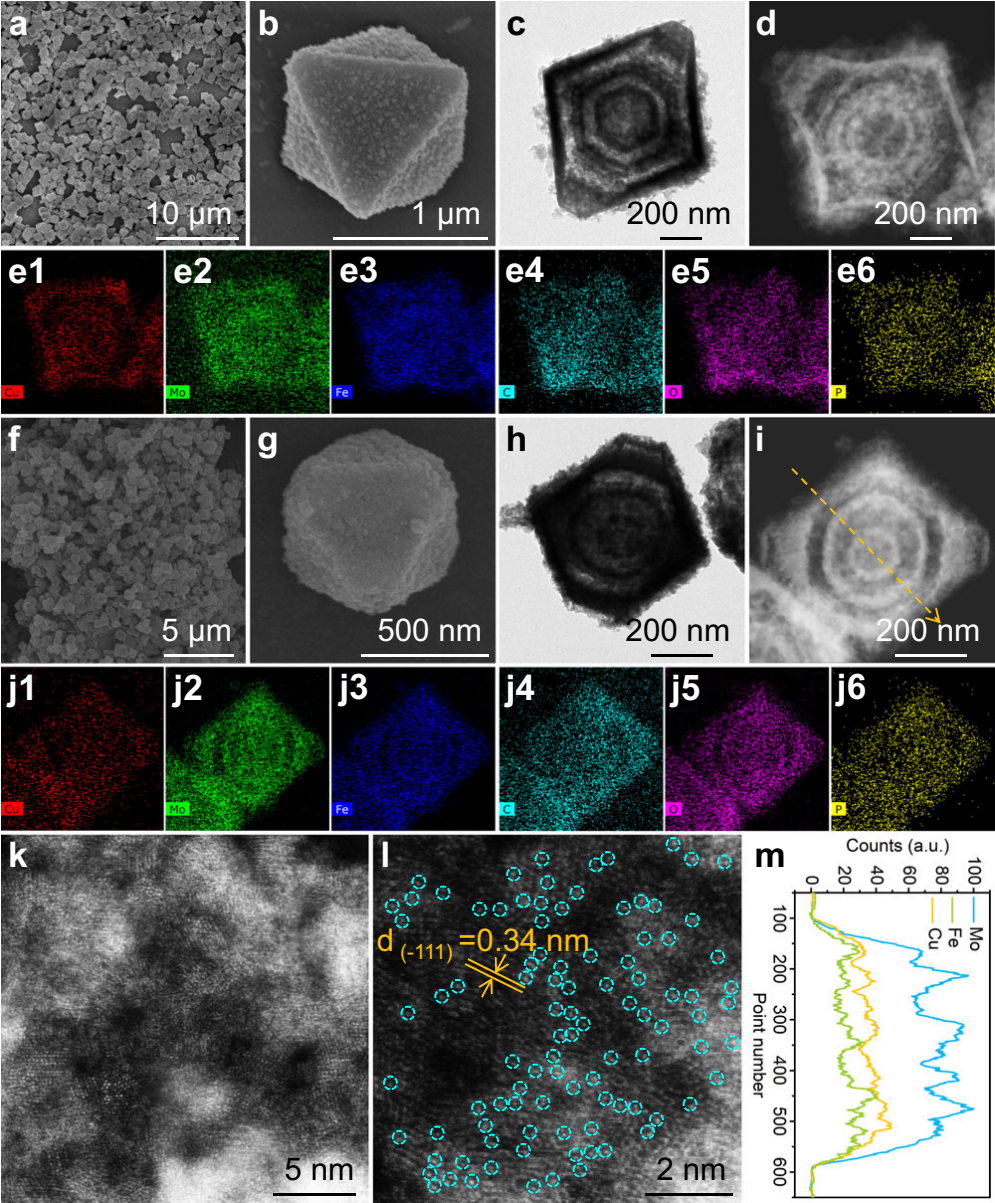
Morphological and structural characterization. **a**, **b** SEM, **c** TEM, **d** HAADF-STEM, and **e1**–**e6** EDX mapping images of Fe-NENU-5. **f**, **g** SEM, **h** TEM, **i** HAADF-STEM, **j1**–**j6** EDX mapping, **k**, **l** AC HAADF-STEM images, and **m** elemental line scan profiles of Cu_4_/Fe_0.3_Mo_0.7_O_2_@C SHHPO.

Fe-NENU-5 retains octahedral morphology of NENU-5 and uniform particle size (Fig. 2a, b) whereas possesses rougher external surfaces. Transmission electron microscopy (TEM) image (Fig. 2c) clearly disclose the triple-shelled hollow nanostructure of Fe-NENU-5 with even shell thickness (60–70 nm), intershell spacings (70 nm) and hollow cavities (250 nm). Further, HAADF-STEM and the corresponding elemental mapping images (Fig. 2d, e) show the homogeneous distribution of Cu, Mo, Fe, C, O, and P elements throughout the Fe-NENU-5. Notably, no obvious Cu or Fe aggregations are observed, excluding their residue or adsorption within the nanostructure. These results indicate that the cation exchange-diffusion process leads to a partial substitution of Fe to Cu, and simultaneously an evolution of the solid NENU-5 octahedron into multi-shelled hollow hierarchical structure with the generation of meso- and macropores inside.

The morphology evolution of NENU-5 nanocrystals is tracked and characterized by SEM and TEM at different periods of the cation exchange-diffusion process (Supplementary Fig. 3a–e). At the very beginning, the Fe^3+^ cations adsorb on the external surfaces of NENU-5 and in situ substitute some Cu nodes, leading to partial decomposition of NENU-5 nanostructure and the resultant rough external surfaces. With time goes by, the Fe^3+^ cations are immerged into the bulk NENU-5 nanocrystals for continuous substitution, resulting in the structural evolution of the solid NENU-5 octahedrons into multi-shelled hollow hierarchical ones within 30 min (Supplementary Fig. 3a–c). With a further increase in etching time (i.e., 50 and 60 min), double- and even single-shelled hollow nanostructures (Supplementary Fig. 3d, e) are formed.

The content of Fe in the discarded solution decreases while that of Cu correspondingly increases in the cation exchange-diffusion process (Supplementary Fig. 4), confirming the partial replacement of Cu to Fe in the material. Besides, the weakened intensity of the NENU-5 diffraction peaks in the XRD patterns of the as-prepared multi-shelled nanostructures (Supplementary Fig. 5a) suggest the partially preserved NENU-5 topologies. In addition, the effects of Fe^3+^ concentration on the morphology of the obtained Fe-NENU-5 are also investigated. A series of cation-exchanging solutions containing 0.05–0.30 g FeCl_3_ ∙ 6H_2_O are prepared for the substitution. Characteristic diffraction peaks of NENU-5 are clearly observed in the XRD patterns of the obtained counterparts (Supplementary Fig. 5b), indicating the partially retained NENU-5 crystalline structure. When the FeCl_3_ ∙ 6H_2_O dosage increases from 0 to 0.30 g, the external surfaces of the as-prepared Fe-NENU-5 gradually turn from smooth to rough (Supplementary Fig. 3f–j). Specifically, a low FeCl_3_ ∙ 6H_2_O loading (<0.05 g) leads to smooth external surfaces and solid yolk-shell nanostructures of Fe-NENU-5 (Supplementary Fig. 3f, j). Rough external surfaces and triple-shelled hollow nanostructures with irregular intershell gaps are observed at the FeCl_3_ ∙ 6H_2_O usage of 0.10 g (Supplementary Fig. 3h). When the FeCl_3_ ∙ 6H_2_O loading is higher than 0.20 g, the triple-shelled nanostructure collapses with apparent generation of defects (Supplementary Fig. 3i, j). These results suggest that a 40 min period of cation exchanging with 0.15 g FeCl_3_ ∙ 6H_2_O loading is appropriate for the formation of hollow triple-shelled nanostructure of Fe-NENU-5 (Fig. 2a–d). An annealing treatment is then carried out under Ar atmosphere to convert Fe-NENU-5 into Cu_4_/Fe_0.3_Mo_0.7_O_2_@C SHHPO. The pyrolysis temperature is set at 600 °C, higher than the decomposition temperatures of NENU-5 and Fe-NENU-5 determined by thermogravimetric analysis (Supplementary Fig. 6).

### Structural characterizations of Cu_4_/Fe_0.3_Mo_0.7_O_2_@C SHHPO

The as-prepared Cu_4_/Fe_0.3_Mo_0.7_O_2_@C SHHPO inherits the octahedral morphology from Fe-NENU-5 but exhibits a shrunken size of ca. 550 nm (Fig. 2f–h). Cu_4_/Fe_0.3_Mo_0.7_O_2_@C SHHPO also features type-IV N_2_-sorption isotherms while with a reduced N_2_ uptake and an enlarged hysteresis loop (Supplementary Fig. 1e) as compared with Fe-NENU-5. Besides, the pore-size distribution curves reveal a widened pore-size distribution in 10–100 nm (Supplementary Fig. 2f), suggesting a large amount of micropores are coalesced into meso- and even macropores during the pyrolysis. Correspondingly, the Brunauer–Emmett–Teller (BET) specific surface area of Cu_4_/Fe_0.3_Mo_0.7_O_2_@C SHHPO is reduced to 169.7 m^2^ g^−1^ with a pore volume of 0.41 cm^3^ g^−1^ (Supplementary Table 1). In comparison, Cu_x_/MoO_2_@C prepared via the direct pyrolysis of NENU-5 has a slightly larger BET specific surface area (182.9 m^2^ g^−1^) with smaller pore volume (0.24 cm^3^ g^−1^) due to the more micropores and fewer mesopores.

The multi-shelled hollow hierarchical nanostructure of Cu_4_/Fe_0.3_Mo_0.7_O_2_@C SHHPO is further verified by TEM (Fig. 2h), showing easily identified triple-shelled nanostructures (Fig. 2i and m) without obvious agglomeration (Fig. 2j1–j6). AC HAADF-STEM images of the ultrafine Mo oxide nanocrystals (Fig. 2k, l) clearly demonstrate the atomic distribution of Fe and Cu species (isolated bright dots) into the MoO_2_ (−1 1 1) lattice planes due to their Z-contrast differences. The support is denoted as Fe_0.3_Mo_0.7_O_2_ after Fe incorporation in which the atom ratio of Fe to Mo is determined by ICP-OES (Supplementary Table 1). No obvious structural changings of MoO_2_ are detected after the atomic doping of Fe. The XRD diffraction peaks of Cu_4_/Fe_0.3_Mo_0.7_O_2_@C match well with those of MoO_2_ (Supplementary Fig. 2d). Besides, no characteristic XRD peaks of Cu composites are observed, which is possibly related to their high dispersion and relatively small size. In contrast, the Cu_x_/MoO_2_@C counterpart shows octahedral morphology with obvious metal agglomerations (Supplementary Fig. 7), indicating the proposed cation exchange-diffusion strategy is essential for the achievement of both multi-shelled hollow hierarchical nanostructure and high metal dispersion.

**Fig. 3:**
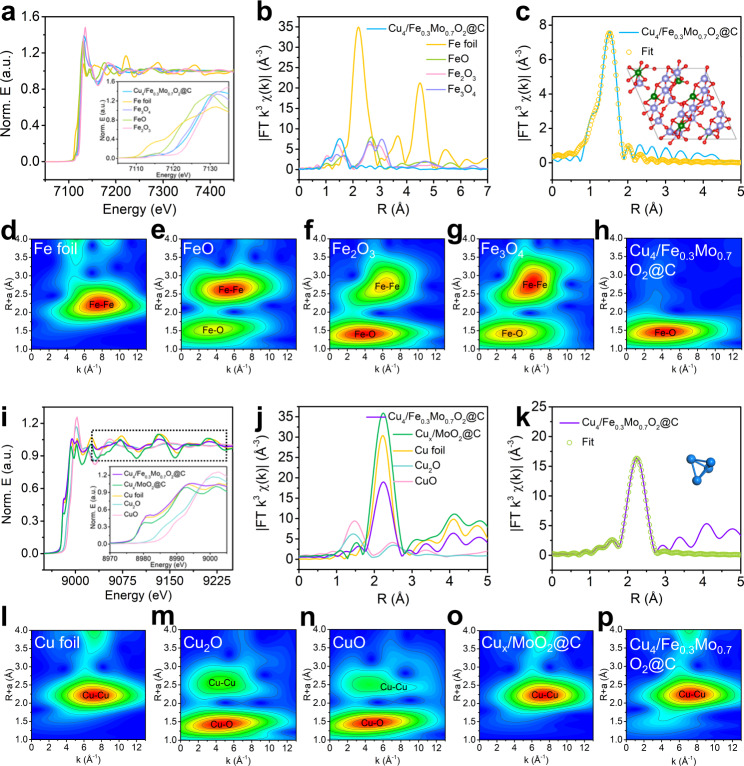
XANES characterization of Cu_4_/Fe_0.3_Mo_0.7_O_2_@C. **a** XANES spectra at the Fe K-edge of Fe foil, FeO, Fe_2_O_3_, Fe_3_O_4_, and Cu_4_/Fe_0.3_Mo_0.7_O_2_@C (Fe). **b** Fourier transform (FT) at the Fe K-edge of Fe foil, FeO, Fe_2_O_3_, Fe_3_O_4_, and Cu_4_/Fe_0.3_Mo_0.7_O_2_@C (Fe), and **c** corresponding EXAFS fitting curves of Cu_4_/Fe_0.3_Mo_0.7_O_2_@C (Fe) in R space. Inset: model of Fe_0.3_Mo_0.7_O_2_; Fe (green), Mo (purple), and O (red). WT for the k^3^-weighted EXAFS signals of **d** Fe foil, **e** FeO, **f** Fe_2_O_3_, **g** Fe_3_O_4_, and **h** Cu_4_/Fe_0.3_Mo_0.7_O_2_@C (Fe). **i** XANES spectra at the Cu K-edge of Cu foil, Cu_2_O, CuO, Cu_x_/MoO_2_@C, and Cu_4_/Fe_0.3_Mo_0.7_O_2_@C (Cu). **j** Fourier transform (FT) at the Cu K-edge of Cu foil, Cu_2_O, CuO, Cu_x_/MoO_2_@C, and Cu_4_/Fe_0.3_Mo_0.7_O_2_@C (Cu), and **k** corresponding EXAFS fitting curves of Cu_4_/Fe_0.3_Mo_0.7_O_2_@C (Cu) in R space. Inset: model of Cu_4_. WT for the k^3^-weighted EXAFS signals of **l** Cu foil, **m** Cu_2_O, **n** CuO, **o** Cu_x_/MoO_2_@C, and **p** Cu_4_/Fe_0.3_Mo_0.7_O_2_@C (Cu).

X-ray absorption fine structure (XAFS) measurements were performed to investigate the coordination environments of Fe and Cu at atomic level. The Fe K-edge X-ray absorption near-edge structure (XANES) spectra of Cu_4_/Fe_0.3_Mo_0.7_O_2_@C SHHPO are shown in Fig. 3a (with Fe foil, FeO, Fe_2_O_3_, and Fe_3_O_4_ as references). The K-edge of Cu_4_/Fe_0.3_Mo_0.7_O_2_@C is located between Fe_3_O_4_ and Fe_2_O_3_, revealing the valent state of Fe species is between Fe(II) and Fe(III). Besides, a prominent peak at ca. 1.5 Å in the Fourier transform (FT) k^3^-weighted extended X-ray absorption fine structure (EXAFS) spectrum of Cu_4_/Fe_0.3_Mo_0.7_O_2_@C contributes similarly to Fe_2_O_3_ and Fe_3_O_4_ (Fig. 3b), which is attributed to the scattering of Fe–O (Supplementary Table 2). Moreover, no apparent peak at ca. 2.2 Å is observed in Cu_4_/Fe_0.3_Mo_0.7_O_2_@C SHHPO, excluding the formation of Fe–Fe bonds and thus confirming the atomic doping of Fe atoms into the MoO_2_ lattice planes.

To acquire the local structural parameters of Fe atom in Cu_4_/Fe_0.3_Mo_0.7_O_2_@C, quantitative EXAFS fitting was carried out (Fig. 3c, Supplementary Fig. 8, Supplementary Table 2). The fitting curves imply that Fe atoms are coordinated to O atoms in Cu_4_/Fe_0.3_Mo_0.7_O_2_@C with a Fe–O bonding length of 1.98 Å. Besides, the wavelet transforms (WT) analysis was performed to verify the above findings (Fig. 3d–h). As compared with the Fe foil, FeO, Fe_2_O_3_, and Fe_3_O_4_ references, the WT maximum at ~4.2 Å^−1^ is assigned to the Fe–O bonds in Cu_4_/Fe_0.3_Mo_0.7_O_2_@C, in consistent with the EXAFS results. According to the structure-sensitive XAFS test and analysis results, the optimized structural model is established (Fig. 3c inset), which suggests the successful introduction of atomic Fe species into the MoO_2_.

**Fig. 4:**
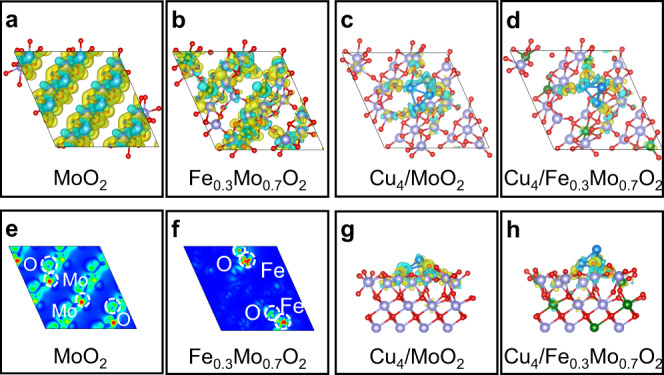
Calculated electronic properties of Cu_4_/Fe_0.3_Mo_0.7_O_2_@C. Top view of the charge densities of **a** MoO_2_ and **b** Fe_0.3_Mo_0.7_O_2_. Top view (**c**, **d**), and side view (**g**, **h**) of the calculated charge transfer difference between subnanometric Cu_4_ cluster and MoO_2_ (**c** and **g**) or Fe_0.3_Mo_0.7_O_2_ (**d** and **h**). 2D charge density distributions of MoO_2_ (**e**) and Fe_0.3_Mo_0.7_O_2_ (**f**). The isosurface value is set to be 0.005 e/Å^3^ and the yellow and cyan areas refer to the charge accumulation and depletion, respectively. Cu (blue), Fe (green), Mo (purple), and O (red).

The effects of atomic Fe doping on the charge distributions over the as-synthesized Fe_0.3_Mo_0.7_O_2_ lattice were investigated by X-ray photoelectron spectroscopy (XPS) and density function theory (DFT) calculations. The characteristic peaks in the Mo *3d* region XPS spectrum of both Cu_4_/Fe_0.3_Mo_0.7_O_2_@C and Cu_x_/MoO_2_@C (Supplementary Fig. 9a, c) reveal the +4 valence state of Mo species^22,23^. After the doping of atomic Fe, an obvious up-shift of ca. 1.0 eV for the characteristic XPS peaks of Mo in Cu_4_/Fe_0.3_Mo_0.7_O_2_@C is observed (Supplementary Figs. 9a, 10a), and the corresponding O *1s* peak is also shifted to higher binding energies (Supplementary Fig. 10b). To exclude the impact of crystal terminations on the XPS spectra, DFT calculations were additionally performed to investigate the electrostatic potential at the surface of Cu_4_/Fe_0.3_Mo_0.7_O_2_@C and Cu_x_/MoO_2_@C models (Supplementary Fig. 11). In comparison with Cu_x_/MoO_2_@C, Cu_4_/Fe_0.3_Mo_0.7_O_2_@C shows significant peak shifts in electrostatic potential and increased work function (5.32 eV vs 4.96 eV). Combining the XPS and DFT results, the observed rigid shift in binding energies is possibly attributed to the electron charge redistributions within the nanocrystals and discrepant surfaces due to the introduction of Fe^24–28^. In addition, the valence states of C, O, and P elements in Cu_4_/Fe_0.3_Mo_0.7_O_2_@C and Cu_x_/MoO_2_@C are also studied by XPS, and the details can be seen in the Supporting Information (Supplementary Figs. 9b, d, 12). Further, remarkable electron accumulations are observed around O atoms (verified by DFT, Fig. 4a, e) in pristine MoO_2_. After Fe doping, the charge density around O atoms is remarkably reduced (Fig. 4b, f), indicating the introduction of Fe leads to a weakened electron accumulation around the neighboring O atoms, which is possibly indexing to the stronger electronegativity of Fe (1.80) than Mo (1.47)^29,30^.

The Cu K-edge XANES and EXAFS spectra were also collected to study the electronic structure and coordination environment of Cu species in Cu_4_/Fe_0.3_Mo_0.7_O_2_@C and Cu_x_/MoO_2_@C (Fig. 3i–p, Supplementary Fig. 13, Supplementary Table 3). The Cu K-edge XANES spectrum of Cu_4_/Fe_0.3_Mo_0.7_O_2_@C is located close to that of Cu foil (Fig. 3i), revealing the metallic state of Cu species^31,32^. A prominent peak at ca. 2.2 Å in the FT k^3^-weighted EXAFS spectrum of Cu_4_/Fe_0.3_Mo_0.7_O_2_@C contributes similarly to Cu foil (Fig. 3j), implying the formation of Cu–Cu bonds, in good agreement with the fitting EXAFS results of R-space spectrum of Cu_4_/Fe_0.3_Mo_0.7_O_2_@C (Fig. 3k)^33–36^. Besides, the intensity maximum at 7 Å^−1^ in the WT contour plots of Cu_4_/Fe_0.3_Mo_0.7_O_2_@C (Fig. 3p) is similar to the Cu foil reference, also suggesting the existence of Cu–Cu bonds. Whereas, the dramatic attenuation of post-edge oscillations (Fig. 3i) suggests the Cu species in Cu_4_/Fe_0.3_Mo_0.7_O_2_@C is lacking of long-range orders^33^. In addition, the sharply reduced first-shell intensity in Cu_4_/Fe_0.3_Mo_0.7_O_2_@C (Fig. 3j) indicates a much lower coordination number of Cu in Cu_4_/Fe_0.3_Mo_0.7_O_2_@C than in Cu foil (6.3 to 12), in consistent with the EXAFS fitting data (Supplementary Table 3). These results suggest the Cu species in Cu_4_/Fe_0.3_Mo_0.7_O_2_@C are present in the form of subnanometric clusters with six Cu–Cu bonds, i.e., Cu_4_, of which the topological nanostructure is illustrated in the inset of Fig. 3k. In comparison, the Cu K-edge XANES spectrum of Cu_x_/MoO_2_@C is located close to that of Cu foil with remarkable post-edge oscillations (Fig. 3i), indicating the metallic state and long-range order of the Cu component (clusters or nanoparticles). Besides, the Cu–Cu coordination number of Cu_x_/MoO_2_@C is ca. 12, implying its relatively larger size of Cu components than that of Cu_4_/Fe_0.3_Mo_0.7_O_2_@C. Considering the coordination number, cluster size and stability, we could infer the Cu component in Cu_4_/Fe_0.3_Mo_0.7_O_2_@C is highly likely to be Cu_4_ clusters with quadrihedron geometry.

Furthermore, in the Cu *2p* XPS spectra of Cu_4_/Fe_0.3_Mo_0.7_O_2_@C and Cu_x_/MoO_2_@C composites (Supplementary Fig. 14), the prominent band locating at 932.5 eV indicates the existence of Cu^0^ species in Cu_x_/MoO_2_@C (the co-existent Cu^2+^ may be related to the surface oxidation). In comparison, Cu^0^ species in Cu_4_/Fe_0.3_Mo_0.7_O_2_@C manifest a slight shift towards higher binding energy by ca. 0.5 eV, possibly caused by the electron transfer from Cu to Fe atoms.

To sum up, the cation exchange-diffusion strategy demonstrated here is effective and precise for the fabrication of Cu_4_/Fe_0.3_Mo_0.7_O_2_@C consisting of Cu subnanometric clusters (four Cu atoms in average) dispersed on ultrafine Fe-doped MoO_2_ nanocrystals (Fe_0.3_Mo_0.7_O_2_). As expected, the atomic doping of Fe into MoO_2_ leads to significantly modified electronic structure of Fe_0.3_Mo_0.7_O_2_ and the consequent charge redistribution inside the obtained Cu_4_ species.

### Catalytic performance of Cu_4_/Fe_0.3_Mo_0.7_O_2_@C SHHPO

The distinctive structure and properties of the Cu_4_/Fe_0.3_Mo_0.7_O_2_@C material inspired us to investigate its catalytic performance. Furfural (FFA) is a commercialized biomass platform molecule, which may be chemically converted into over 80 kinds of valuable chemicals/biofuels^37–40^. One of the most promising strategies for FFA valorization is the aldol condensation of FFA with aldehydes/ketones (e.g., acetone) for the production of α,β-unsaturated aldehydes/ketones, which are well-known as critical intermediates for the production of aviation biofuels (C_8_~C_15_ alkanes) via a hydrodeoxygenation process^41–44^. From a green, economic, and sustainable viewpoint, the direct reaction of saturated monobasic alcohols with FFA may be the most preferred and promising, due to the availability and low cost of alcohols in comparison with their oxidation products such as aldehydes and ketones.

**Fig. 5:**
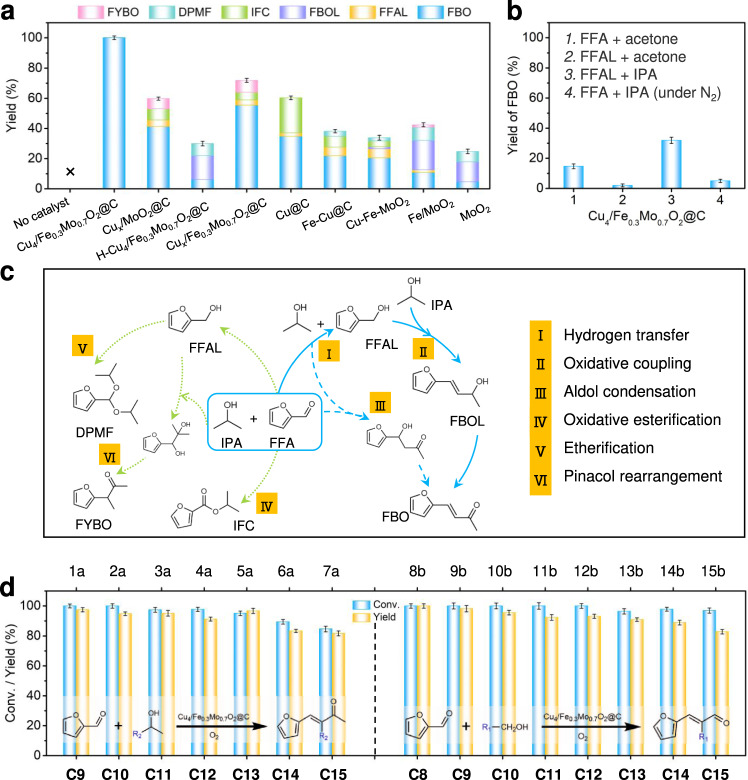
Catalytic performance of Cu_4_/Fe_0.3_Mo_0.7_O_2_@C. **a** Oxidative coupling of FFA with IPA into FBO over different catalysts. Reaction conditions: FFA (0.5 mmol), catalysts (Cu, 3.6 mol%), K_2_CO_3_ (0.1 mmol), IPA (5 mL), O_2_ (2 bar), 120 °C, 16 h. Conversion and yield were determined by GC-MS. For the control catalysts without Cu species, the catalyst usage is 0.03 g. **b** Control experiments using FFA/FFAL and/or IPA/acetone as substrates over Cu_4_/Fe_0.3_Mo_0.7_O_2_@C. **c** Proposed reaction routes and possible by-products. **d** Oxidative coupling of FFA with secondary and primary alcohols (1a-7a, 8b-15b) into C_8_~C_15_ aviation biofuel intermediates over Cu_4_/Fe_0.3_Mo_0.7_O_2_@C. The error bars represent standard deviation based on three measurements.

In this work, we developed a new reaction route for the production of α,β-unsaturated ketones from FFA, i.e., the oxidative coupling of FFA with secondary alcohols. The one-pot cascade oxidative coupling of FFA with isopropanol (IPA) into 4-(2-furyl)-buten-2-one (FBO, Supplementary Fig. 15) is selected as a model reaction. Currently, FBO is produced from the normal aldol condensation of FFA with acetone that is known as a toxic and dangerous solvent. Typically, the reaction is carried out at 120 °C under O_2_ using IPA as both the reactant and solvent, and the results are summarized in Fig. 5a and Supplementary Table 4. The FFA conversion is below the detecting limit in the absence of catalyst (Fig. 5a, Supplementary Table 4, entry 1), suggesting its essential role to produce FBO under the investigated reaction conditions. To our delight, Cu_4_/Fe_0.3_Mo_0.7_O_2_@C exhibits super catalytic performance with 100% FFA conversion and ˃99% FBO yield within 16 h (Fig. 5a, Supplementary Table 4, entry 2).

In order to verify the essential roles of the Cu_4_ clusters and atomic Fe doping into the MoO_2_ support, some related counterparts (e.g., H-Cu_4_/Fe_0.3_Mo_0.7_O_2_@C, Cu_x_/Fe_0.3_Mo_0.7_O_2_@C, Cu-Fe-MoO_2_, Cu@C, Fe-Cu@C, Fe/MoO_2_, and MoO_2_) are also synthesized (Supplementary Figs. 16–19 and Supplementary Table 1) and employed as catalysts in this reaction. These samples afford low to moderate FFA conversions (24.8–71.9%) and FBO yields (4.7–55.5%) (Fig. 5a, and Supplementary Table 4, entries 3–10). H-Cu_4_/Fe_0.3_Mo_0.7_O_2_@C and Cu_x_/Fe_0.3_Mo_0.7_O_2_@C afford moderate catalytic conversions (30.0% and 71.9%, respectively, Fig. 5a, and Supplementary Table 4, entries 4–5).

## Discussion

### Reaction mechanism investigation

On the basis of the intermediates detected in the reaction, the plausible reaction routes are illustrated in Fig. 5c. Initially, FFA is transfer-hydrogenated by IPA into furfuryl alcohol (FFAL) with the generation of equivalent amount of acetone (route I). The obtained FFAL subsequently reacts with another IPA (greatly excessive) molecule through an oxidative-coupling process to produce FBOL (route II), which is finally oxidized into FBO. Another possible reaction route is the conventional aldol condensation of FFA with acetone (route III)^45^, which is also investigated as a potential route in the following reaction mechanism.

Some control experiments are carried out to verify the proposed reaction routes (Fig. 5b and Supplementary Table 5). When acetone is employed as the reactant, <15% FBO is obtained (Fig. 5b, columns 1 and 2). Similarly, when the reaction intermediate FFAL is used as the reactant, only 31.9% FBO yield is obtained (Fig. 5b, column 3). These results indicate the FFA transformation over Cu_4_/Fe_0.3_Mo_0.7_O_2_@C is started with the transferring hydrogenation of FFA with IPA, and FFAL could hardly react with acetone under the investigated conditions. In addition, when the reaction atmosphere is changed from O_2_ to N_2_, only 5% FBO yield is detected (Fig. 5b, column 4), revealing that the oxidative coupling step (route II) is the dominant route to produce FBO over the Cu_4_/Fe_0.3_Mo_0.7_O_2_@C using FFA and IPA as the substrates.

**Fig. 6:**
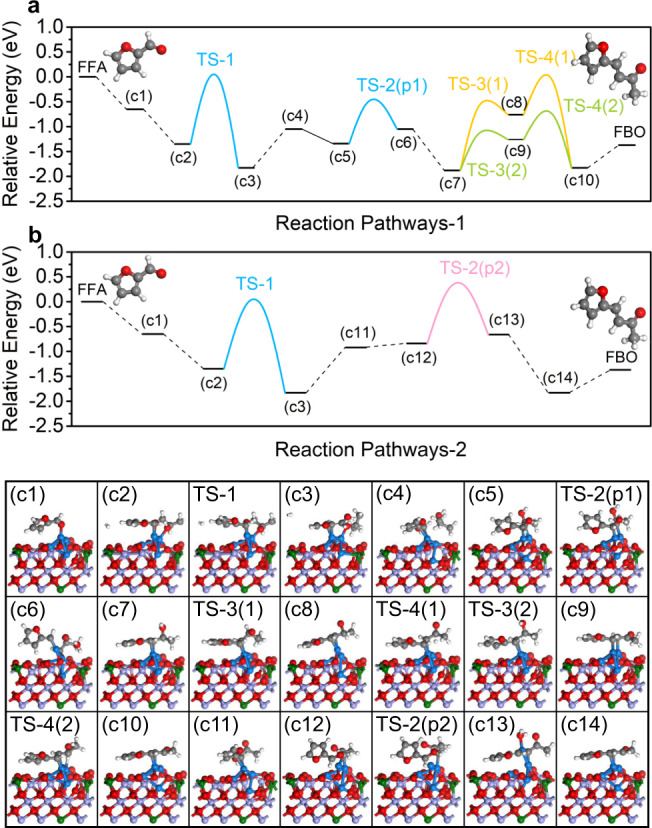
The plausible reaction mechanism over Cu_4_/Fe_0.3_Mo_0.7_O_2_@C. **a**, **b** The free energy diagram for oxidative coupling of FFA with IPA, and **c**1–**c**14 the simplified surface structures of various reaction species along the reaction pathway on Cu_4_/Fe_0.3_Mo_0.7_O_2_@C. Cu (blue), Fe (green), Mo (purple), O (red), C (gray), and H (white). “TS” denotes a transition state.

DFT calculations were performed to deeply elucidate and bring theoretical insight to the reaction mechanism (Figs. 4, 6, and Supplementary Figs. 20, 21). The differential charge densities of Cu_4_/MoO_2_ (Fig. 4c, g) and Cu_4_/Fe_0.3_Mo_0.7_O_2_ (Fig. 4d, h) are simulated and optimized. In Cu_4_/MoO_2_, uniform electron transferring from Cu_4_ subnanometric clusters to MoO_2_ support is observed (averagely ca. 0.175 e for each Cu atom). Interestingly, after Fe doping, the electron transferring between Cu_4_ subnanometric clusters and Fe_0.3_Mo_0.7_O_2_ is modified, each Cu atom at the bottom of Cu_4_ cluster donates ca. 0.2 e towards the Fe_0.3_Mo_0.7_O_2_. In terms of the top Cu atom in Cu_4_ (denoted as Cu^0^), the electron transferring is negligible (verified by Bader charge analysis in Supplementary Table 8). As a result, the symmetric charge distribution inside Cu_4_ clusters is redistributed after the atomic doping of Fe upon MoO_2_, which is believed largely contributing to the observed outstanding catalytic performance of Cu_4_/Fe_0.3_Mo_0.7_O_2_@C in the FFA oxidative coupling reaction.

A plausible reaction mechanism is proposed for the oxidative coupling of FFA with IPA to FBO over Cu_4_/Fe_0.3_Mo_0.7_O_2_@C (Fig. 6, for details, see Supplementary Figs. 20, 21). The reaction pathway-1 consists of ten elementary steps. At the very beginning, one FFA molecule bonds with the Cu^0^ atom at the top of Cu_4_ to form (c1) due to a relatively low binding energy (−0.65 eV). Subsequently, the adsorbed FFA molecule reacts with one adsorbed IPA molecule (c2), overcoming the energy barrier of 1.47 eV (TS-1) and forming FFAL radical and one acetone molecule (illustrated as c3). Afterwards, the generated FFAL radical (c4) further reacts with another IPA molecule to form a (c5) complex, which undergoes a dehydration process with an energy barrier of 0.92 eV (TS-2(p1)) to produce *ph-CHCH-CH(OH)-CH_3_ (c6-c7, the ph represents the furan ring). Afterwards, the *ph-CHCH-CH(OH)-CH_3_ undergoes a dehydrogenation reaction which tends to take place on the carbon side (to form c9) than at oxygen side (to form c8) due to the former’s relatively low energy barrier (0.82 eV in comparison with 1.43 eV). Then, another dehydrogenation reaction occurs on the oxygen side overcoming an energy barrier of 0.61 eV (TS-4) to produce *FBO (c10), which at last desorbs endothermically (0.46 eV) from the catalyst to yield FBO. In addition, the enthalpies and barriers for the first few steps of the reaction mechanism over the Cu_4_/MoO_2_@C were also calculated to interpret the impact of Fe substitution. DFT results (Supplementary Fig. 22) reveal a much higher energy barrier (TS-1) over Cu_4_/MoO_2_ than Cu_4_/Fe_0.3_Mo_0.7_O_2_ (1.65 eV vs 1.47 eV) within the first few steps, suggesting the atomic Fe species can indeed facilitate the reaction process through regulating the electronic properties.

After the first three elementary steps of pathway-1, the generated acetone may react with the residue FFA. Therefore, the reaction mechanism of FFA and the generated acetone are additionaly investigated in pathway-2. In the fourth elementary step, the energy barrier of the reaction between FFAL radical and acetone to produce (c11) is much higher in comparison with that of pathway-1 (0.91 to 0.78 eV). In addition, the subsequent dehydration reaction (from c12 to c13, TS-2(p2)) requires an energy barrier as high as 1.27 eV, also higher than that of the reaction pathway-1 (0.82–0.92 eV). Therefore, the reaction pathway-2 (the generated acetone react with FFA to give FBO) is not preferential in both thermodynamic and kinetic ways as compared with the reaction pathway-1.

To investigate the general applicability of Cu_4_/Fe_0.3_Mo_0.7_O_2_@C, C_3_~C_10_ secondary and primary alcohols were also employed to synthesize the corresponding C_8_~C_15_ ketones and aldehydes as aviation biofuel intermediates under the optimized reaction conditions (Fig. 5d, Supplementary Tables 6, 7). Good to excellent yields (81.7–100%) of the C_8_~C_15_ ketones and aldehydes are achieved through slightly modifying the reaction conditions (e.g., extending the reaction time or increasing the reaction temperature). These reaction results demonstrate the high practical potentials of Cu_4_/Fe_0.3_Mo_0.7_O_2_@C in the valorization of the biomass-derived FFA to produce biofuels.

After reaction, the catalyst was easily isolated from the reaction mixture and directly reused after washing and drying. No obvious activity loss is observed in the continuous recycling tests (Supplementary Fig. 23a), implying the good stability and recyclability of Cu_4_/Fe_0.3_Mo_0.7_O_2_@C. Besides, the Cu, Fe and Mo contents in the reaction solution collected by hot filtration (Supplementary Fig. 23b) are below the detecting limits of ICP-OES, indicating the loss of metals is negligible, which could account for the maintenance of catalytic activity. Furthermore, XRD (Supplementary Fig. 24f) and XPS (Supplementary Fig. 24i–k) results confirm the well-preserved crystallization and composition of the Cu_4_/Fe_0.3_Mo_0.7_O_2_@C. TEM (Supplementary Fig. 24a), HAADF-STEM (Supplementary Fig. 24b), AC HAADF-STEM (Supplementary Fig. 24c), the corresponding EDX mapping (Supplementary Fig. 24d) images and elemental line scan profiles (Supplementary Fig. 24e) and N_2_ adsorption–desorption isotherms (Supplementary Fig. 24g, h) demonstrate the well-retained multi-shelled hollow hierarchical nanostructure consisting of Cu_4_ clusters on the Fe-doped MoO_2_ (Cu_4_/Fe_0.3_Mo_0.7_O_2_@C).

In summary, we have demonstrated that the electronic property of M_x_ in SCC can be efficiently modulated through atomically decorating the support. As an example, subnanometric Cu clusters are immobilized onto Fe-doped MoO_2_ support to fabricate the Cu_4_/Fe_0.3_Mo_0.7_O_2_@C nanocomposite. Systematical characterizations reveal the uniform subnanometric Cu clusters composing of four Cu atoms in average, atomically Fe-doped MoO_2_ support and distinctive metal-support interactions between them, which eventually lead to charge redistribution inside the subnanometric Cu clusters. The obtained Cu_4_/Fe_0.3_Mo_0.7_O_2_@C shows excellent catalytic performance in the newly developed reaction route of FFA to FBO, i.e., oxidative coupling of FFA with IPA, outperforming the conventionally prepared counterparts. DFT and control experiments uncover the reaction pathways and suggest the superior catalytic performance of Cu_4_/Fe_0.3_Mo_0.7_O_2_@C is mostly originated from the charge redistribution inside the Cu clusters. Moreover, this catalyst is also highly efficient for the preparation of C_8_~C_15_ aviation biofuel intermediates using the corresponding secondary and even primary alcohols as feedstock. The proposed strategy of modulating the electronic structures of metal single clusters through atom-decoration of support may present a new dimension for the development of advanced catalysts with precisely adjusted electronic properties for various frontier applications.

## Methods

### Synthesis of NENU-5 truncated octahedron

All chemicals were purchased from commercial sources and directly used without further purification. In a typical synthesis, Cu(OAc)_2_ ∙ H_2_O (1 mmol, 0.2 g), L-glutamic acid (1.02 mmol, 0.074 g), and H_3_PMo_12_O_40_ (0.3 g) were dissolved in 40 mL deionized water. After stirring for 1 h under ambient condition, a 40 mL ethanol solution containing H_3_BTC (0.67 mmol, 0.14 g) was added to the above solution under vigorous stirring. After stirring for another 14 h, the resulting green precipitates were collected by centrifugation, washed with deionized water and ethanol, and dried under vacuum at 50 °C overnight.

### Synthesis of Fe-NENU-5

Typically, the as-synthesized NENU-5 (0.05 g) was dispersed into a 60 mL aqueous solution containing FeCl_3_ ∙ 6H_2_O (0.15 g). After stirring for 40 min, the resulting yellow-green precipitates were collected by centrifugation, washed twice with deionized water and ethanol, and dried under vacuum at 50 °C overnight to obtain Fe-NENU-5.

### Synthesis of Cu_4_/Fe_0.3_Mo_0.7_O_2_@C, H-Cu_4_/Fe_0.3_Mo_0.7_O_2_@C, and Cu_x_/Fe_0.3_Mo_0.7_O_2_@C

Typically, the as-prepared Fe-NENU-5 was placed in a tubular furnace (BTF-1200C, Anhui BEQ Equipment Technology Co., Ltd.) and heated to 600 °C at a ramp rate of 1 °C min^−1^ and kept for 120 min under Ar atmosphere to yield Cu_4_/Fe_0.3_Mo_0.7_O_2_@C. The obtained Cu_4_/Fe_0.3_Mo_0.7_O_2_@C powders were thoroughly washed with Fe^3+^ aqueous solution and deionized water to partially remove Cu species and yield H-Cu_4_/Fe_0.3_Mo_0.7_O_2_@C. The obtained Cu_4_/Fe_0.3_Mo_0.7_O_2_@C powders were immersed into Cu(OAc)_2_ ∙ H_2_O aqueous solution and then heated to 600 °C at a ramp rate of 1 °C min^−1^ and kept for 120 min under Ar atmosphere to obtain Cu_x_/Fe_0.3_Mo_0.7_O_2_@C.

### Synthesis of Cu_x_/MoO_2_@C

Typically, the as-prepared NENU-5 was placed in a tubular furnace (BTF-1200C, Anhui BEQ Equipment Technology Co., Ltd.) and heated to 600 °C at a ramp rate of 1 °C min^−1^ and kept for 120 min under Ar atmosphere to yield Cu_x_/MoO_2_@C.

### Synthesis of Cu-BTC and Cu@C

In a typical synthesis, Cu(OAc)_2_ ∙ H_2_O (1 mmol, 0.2 g) was dissolved in 40 mL deionized water. After stirring for 1 h under ambient condition, a 40 mL ethanol solution containing H_3_BTC (0.67 mmol, 0.14 g) was added to the above solution under vigorous stirring. After stirring for 14 h, the resulting blue precipitates were collected by centrifugation, washed twice with deionized water and ethanol, respectively, and finally dried under vacuum at 50 °C overnight to obtain Cu-BTC. The as-prepared Cu-BTC was placed in a tubular furnace (BTF-1200C, Anhui BEQ Equipment Technology Co., Ltd.), which was heated to 600 °C at a ramp rate of 1 °C min^−1^ and kept for 120 min under Ar atmosphere to yield Cu@C.

### Synthesis of Fe-Cu-BTC and Fe-Cu@C

The as-synthesized Cu-BTC (0.05 g) was dispersed into a 60 mL aqueous solution containing FeCl_3_ ∙ 6H_2_O (0.15 g). After stirring for 40 min, the resulting precipitates were collected by centrifugation, washed twice with deionized water and ethanol, respectively, and finally dried under vacuum at 50 °C overnight to yield Fe-Cu-BTC. The as-prepared Fe-Cu-BTC was placed in a tubular furnace (BTF-1200C, Anhui BEQ Equipment Technology Co., Ltd.), which was heated to 600 °C at a ramp rate of 1 °C min^−1^ and kept for 120 min under Ar atmosphere to yield Fe-Cu@C.

### Synthesis of Fe/MoO_2_

In a typical synthesis, the commercial MoO_2_ (3.75 g) was added to 60 mL aqueous solution containing FeCl_3_ ∙ 6H_2_O (0.15 g) under vigorous stirring. After stirring for 14 h under ambient condition, the resulting precipitates were collected and then heated to 600 °C at a ramp rate of 1 °C min^−1^ and kept for 120 min under Ar atmosphere to yield Fe/MoO_2_.

### Synthesis of Cu-Fe-MoO_2_

In a typical synthesis, Cu(OAc)_2_ ∙ H_2_O (0.1 g), FeCl_3_ ∙ 6H_2_O (0.08 g), and 2.0 g commercial MoO_2_ were added to 50 mL deionized water. The mixture was dried and subsequently subjected to pyrolysis at 600 °C with a ramp rate of 1 °C·min^−1^ and kept for 120 min under Ar atmosphere to yield Cu-Fe-MoO_2_.

### Materials characterization

The size and morphology of materials were studied by scanning electron microscopy (SEM) and transmission electron microscopy (TEM). SEM was carried out on a JEOL-6700 instrument. High-angle annular dark-field scanning transmission electron microscopy (HAADF-STEM) and spherical aberration correction HAADF-STEM (AC HAADF-STEM) were recorded on a FEI Titan Cubed Themis G2 300S/TEM with a probe corrector and a monochromator at 200 kV. Powder X-ray diffraction (PXRD) patterns of the samples were obtained on a Rigaku diffractometer (D/max-IIIA, 3 kW) with Cu K_α_ radiation (*λ* = 1.5406 Å) at a voltage of 40 kV and a current of 10 mA at room temperature. Brunauer–Emmett–Teller (BET) surface area and pore-size measurements were performed on a Micromeritics ASAP 2020 M instrument at 77 K. Before the analysis, the samples were degassed at 50 °C for 12 h. X-ray photoelectron spectroscopy (XPS) was collected on a Thermo Scientific K-Alpha system with the C *1s* peak (284.6 eV) as reference. The metal contents of the samples were determined by ICP-OES on an Optima 8300 instrument. The C and O elemental contents of the samples were measured on a Euro Vector EA3000 instrument. Thermogravimetric analysis (TGA) was performed on a NETZSCH STA449C instrument loaded with 5 mg sample using a heating rate of 5 °C/min under argon atmosphere. The X-ray absorption experiments were carried out at the XAS station (BL14W1) of the Shanghai Synchrotron Radiation Facility (SSRF). The electron storage ring was operated at 3.5 GeV. Si (311) double-crystal was used as the monochromator, and the data was collected using solid-state detector under ambient conditions. The beam size was limited by the horizontal and vertical slits with the area of 1 × 4 mm^2^ during XAS measurements.

### Calculation details

All the calculations are performed in the framework of the density functional theory with the projector augmented plane-wave method, as implemented in the Vienna ab initio simulation package. The generalized gradient approximation proposed by Perdew, Burke, and Ernzerhof is selected for the exchange-correlation potential. The cut-off energy for plane wave is set to 400 eV. The energy criterion is set to 10^−5 ^eV in iterative solution of the Kohn-Sham equation. All the structures are relaxed until the residual forces on the atoms have declined to <0.05 eV/Å. The weak interaction was described by DFT + D3 method using empirical correction in Grimme’s scheme. The vacuum space was set to be more than 12 Å, which was enough to avoid the interaction between periodical images. For MoO_2_, a four layer of (−1 1 1) surface was used, and 33% Mo atoms was replaced by Fe for the structure of Fe-doped MoO_2_. The Brillouin zones of structures were sampled with 3 × 3 × 1 k points. The minimum energy pathway for transition state searching process was determined by using a climbing image nudged elastic band method (CINEB).

### One-pot cascade oxidative coupling of FFA with IPA

The one-pot cascade oxidative coupling of FFA with IPA was carried out in a 25 mL high-pressure reactor (NSG25-P5-T3-SS1-SV, Anhui Chem-n Instrument Co., Ltd.) equipped with a magnetic stirrer. In a typical run, FFA (0.5 mmol), catalyst (Cu 3.6 mol% relative to FFA), K_2_CO_3_ (0.1 mmol), and IPA (5 mL) were sealed in the reactor. The reactor was evacuated, refilled with 2 bar O_2_, and heated to 120 °C for 16 h under magnetic stirring. After reaction, the reactor was cooled to room temperature. The catalyst was isolated from the solution by centrifugation and directly reused after washing and drying. The product was quantified by a GC-MS spectrometer (Agilent, 7890B GC/5977A MS) equipped with a DB-35 MS UI capillary column (0.25 mm × 30 m).

The conversions and selectivities were calculated using the following equations:

1 $${{{{{\rm{FFA}}}}}}\; {{{{{\rm{conversion}}}}}}=\left(1-\frac{{{{{{\rm{Moles}}}}}}\; {{{{{\rm{of}}}}}}\; {{{{{\rm{FFA}}}}}}}{{{{{{\rm{Moles}}}}}}\; {{{{{\rm{of}}}}}}\; {{{{{\rm{FFA}}}}}}\; {{{{{\rm{loaded}}}}}}}\right)\times 100 \%$$

2 $${{{{{\rm{FBO}}}}}}\; {{{{{\rm{selectivity}}}}}}=\left(\frac{{{{{{\rm{Moles}}}}}}\; {{{{{\rm{of}}}}}}\; {{{{{\rm{FBO}}}}}}}{{{{{{\rm{Moles}}}}}}\; {{{{{\rm{of}}}}}}\; {{{{{\rm{FFA}}}}}}\; {{{{{\rm{converted}}}}}}}\right)\times 100 \%$$

3 $${{{{{\rm{FBO}}}}}}\; {{{{{\rm{yield}}}}}}=\left(\frac{{{{{{\rm{Moles}}}}}}\; {{{{{\rm{of}}}}}}\; {{{{{\rm{FBO}}}}}}}{{{{{{\rm{Moles}}}}}}\; {{{{{\rm{of}}}}}}\; {{{{{\rm{FFA}}}}}}\; {{{{{\rm{loaded}}}}}}}\right)\times 100 \%$$

## Supplementary information

Supplementary Information

## Data Availability

The authors declare that the data supporting the findings of this study are available within the paper and its Supplementary Information files.
