# Supplementary material for: Subnanometric Cu clusters on atomically Fe-doped MoO2 for furfural upgrading to aviation biofuels
Source: Nat Commun. 2022 May 11;13:2591. doi: 10.1038/s41467-022-30345-0 (PMC9095587; doi:10.1038/s41467-022-30345-0)
Supplement: Supplementary file 1 — Supplementary Information [file 41467_2022_30345_MOESM1_ESM.pdf]

Supplementary Information for

Subnanometric Cu clusters on atomically Fe-doped MoO_2 for furfural upgrading to aviation biofuels

Xin Zhao[1], Fengliang Wang[1], Xiangpeng Kong[2], Ruiqi Fang[1,*], Yingwei Li[1,3,*]

[1]State Key Laboratory of Pulp and Paper Engineering, School of Chemistry and Chemical Engineering, South China University of Technology, Guangzhou 510640, China.

Email: fangrq@scut.edu.cn; liyw@scut.edu.cn

[2]The School of Materials Science and Engineering, Harbin Institute of Technology, Shenzhen 518055, China.

[3]South China University of Technology-Zhuhai Institute of Modern Industrial Innovation, Zhuhai 519175, China.

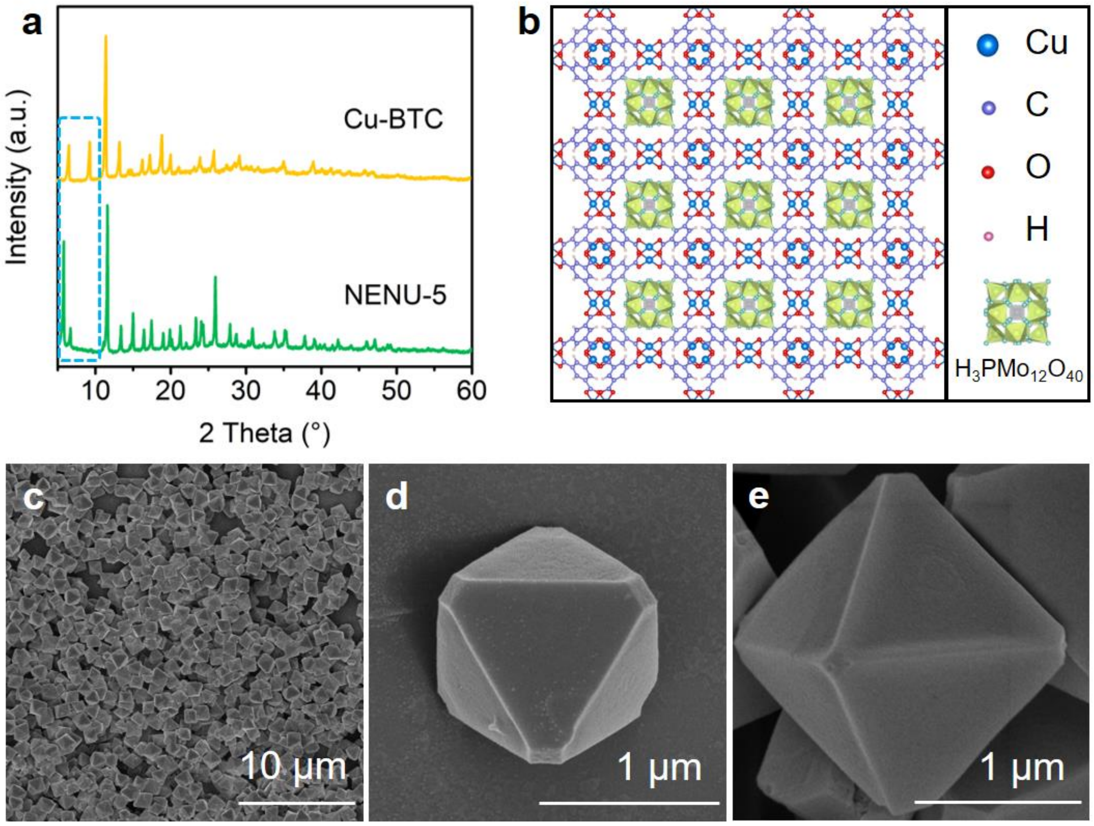

Supplementary Figure 1. Characterizations of NENU-5 and Cu-BTC. (a) XRD patterns of NENU-5 (with $H_3PMo_{12}O_{40}$) and Cu-BTC (without $H_3PMo_{12}O_{40}$). (b) The detailed structure of NENU-5. (C-E) SEM images of NENU-5. NENU-5 and Cu-BTC exhibit different diffraction peaks especially below 10°, which may be attributed to the introduction of $H_3PMo_{12}O_{40}$ into the pores of Cu-BTC.

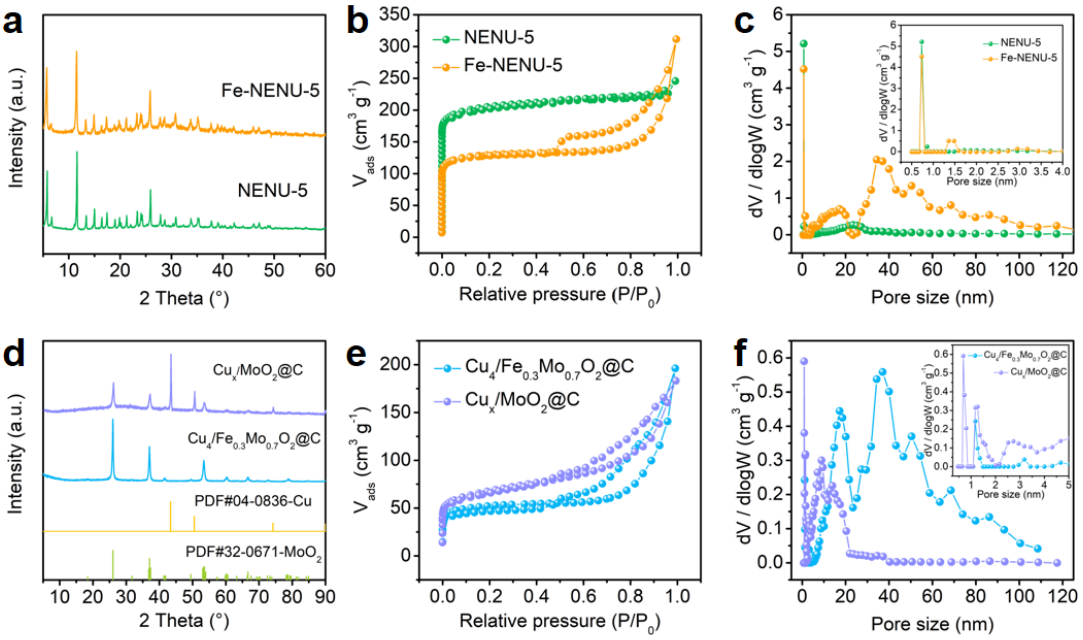

Supplementary Figure 2. Characterizations of NENU-5 and Fe-NENU-5. (a) XRD patterns, (b) N_2 adsorption-desorption isotherms, and (c) corresponding pore-size distributions of NENU-5 and Fe-NENU-5. (d) XRD patterns, (e) N_2 adsorption-desorption isotherms, and (f) corresponding pore-size distributions of $Cu_4/Fe_{0.3}Mo_{0.7}O_2@C$ and $Cu_x/MoO_2@C$.

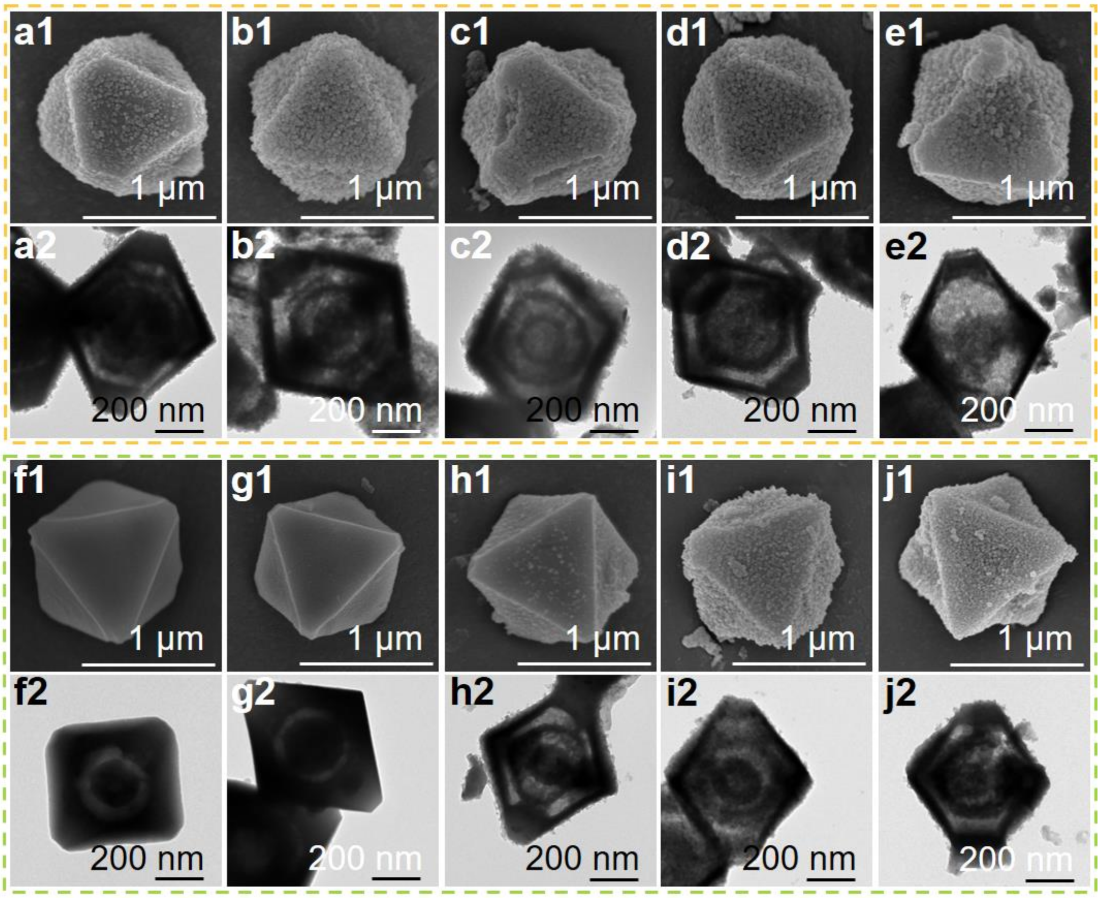

Supplementary Figure 3. SEM and TEM images of the resultant materials after NENU-5 etching. (a1-e1) SEM images, (a2-e2) TEM images of the corresponding materials obtained by etching of NENU-5 in 60 mL of $FeCl_3 \cdot 6H_2O$ (0.15 g) aqueous solution for different time: (a) 10 min, (b) 20 min, (c) 30 min, (d) 50 min, and (e) 60 min. (f1-j1) SEM images, (f2-j2) TEM images of the corresponding materials obtained by etching of NENU-5 in 60 mL of $FeCl_3 \cdot 6H_2O$ aqueous solution for 40 min with different dosage: (f) 0, (g) 0.05 g, (h) 0.10 g, (i) 0.20 g, and (j) 0.30 g.

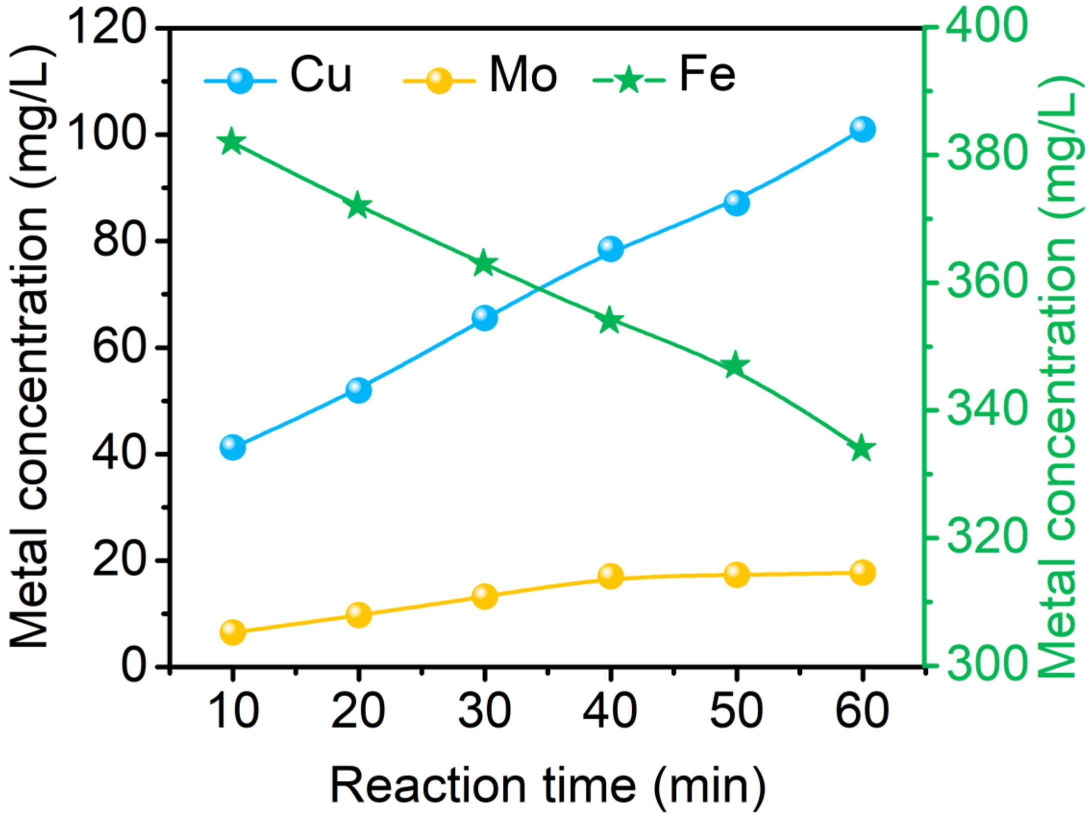

Supplementary Figure 4. The metal concentrations (measured by ICP-OES) in the Fe^{3+} (0.15 g/60 mL) etching solution after reaction for different time.

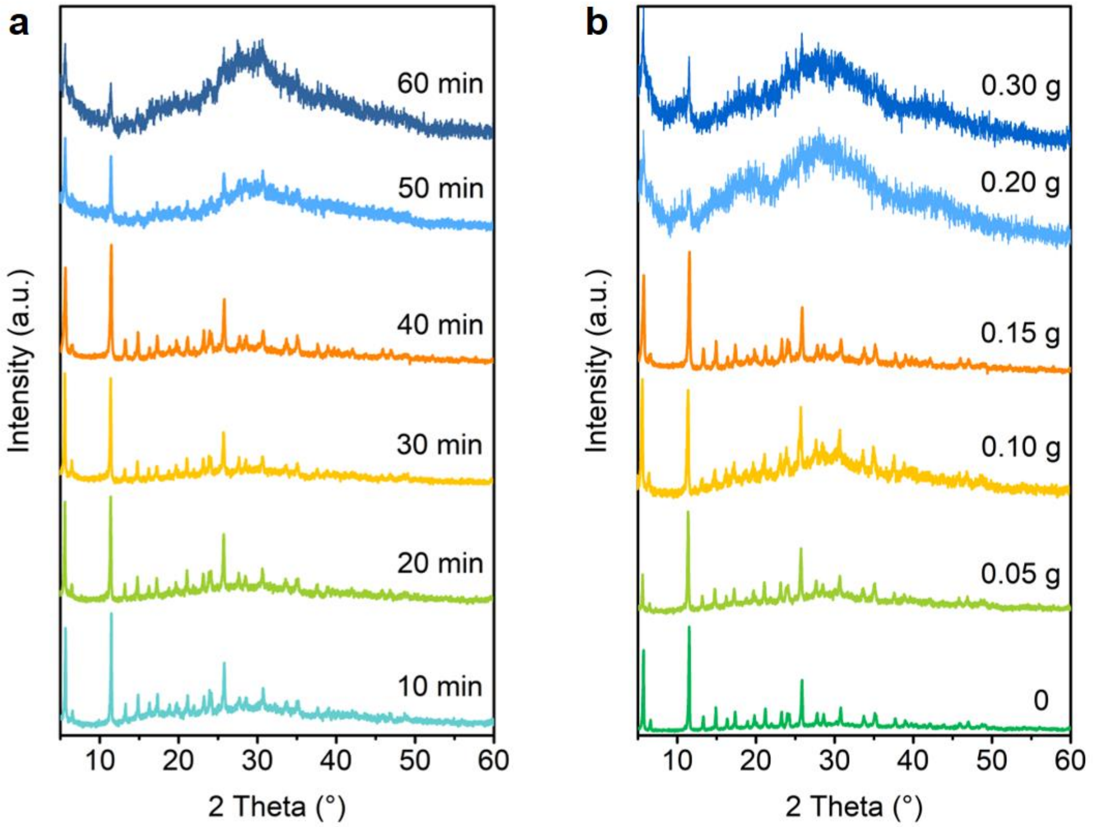

Supplementary Figure 5. XRD patterns of the resultant Fe-NENU-5 after etching. (a) obtained by etching of NENU-5 in 60 mL $FeCl_3 \cdot 6H_2O$ (0.15 g) aqueous solution for different time: 10 min, 20 min, 30 min, 40 min, 50 min, and 60 min; (b) obtained by etching of NENU-5 in 60 mL $FeCl_3 \cdot 6H_2O$ aqueous solution for 40 min with different dosage: 0, 0.05 g, 0.10 g, 0.15 g, 0.20 g, and 0.30 g.

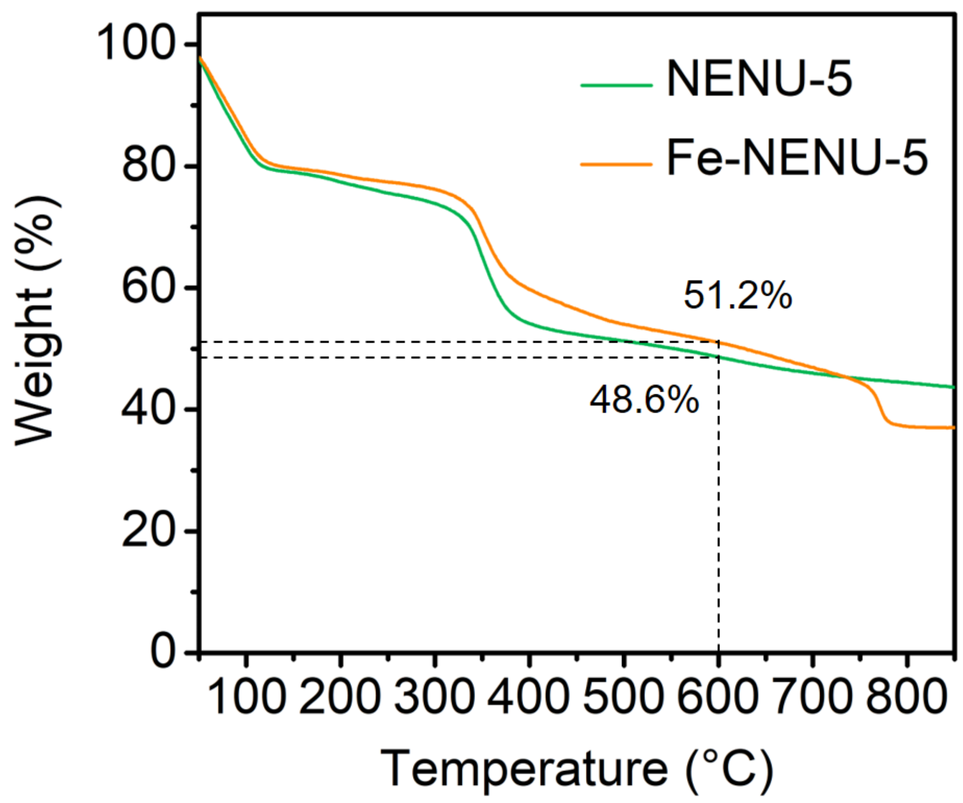

Supplementary Figure 6. TG curves of NENU-5 and Fe-NENU-5 under Ar flow.

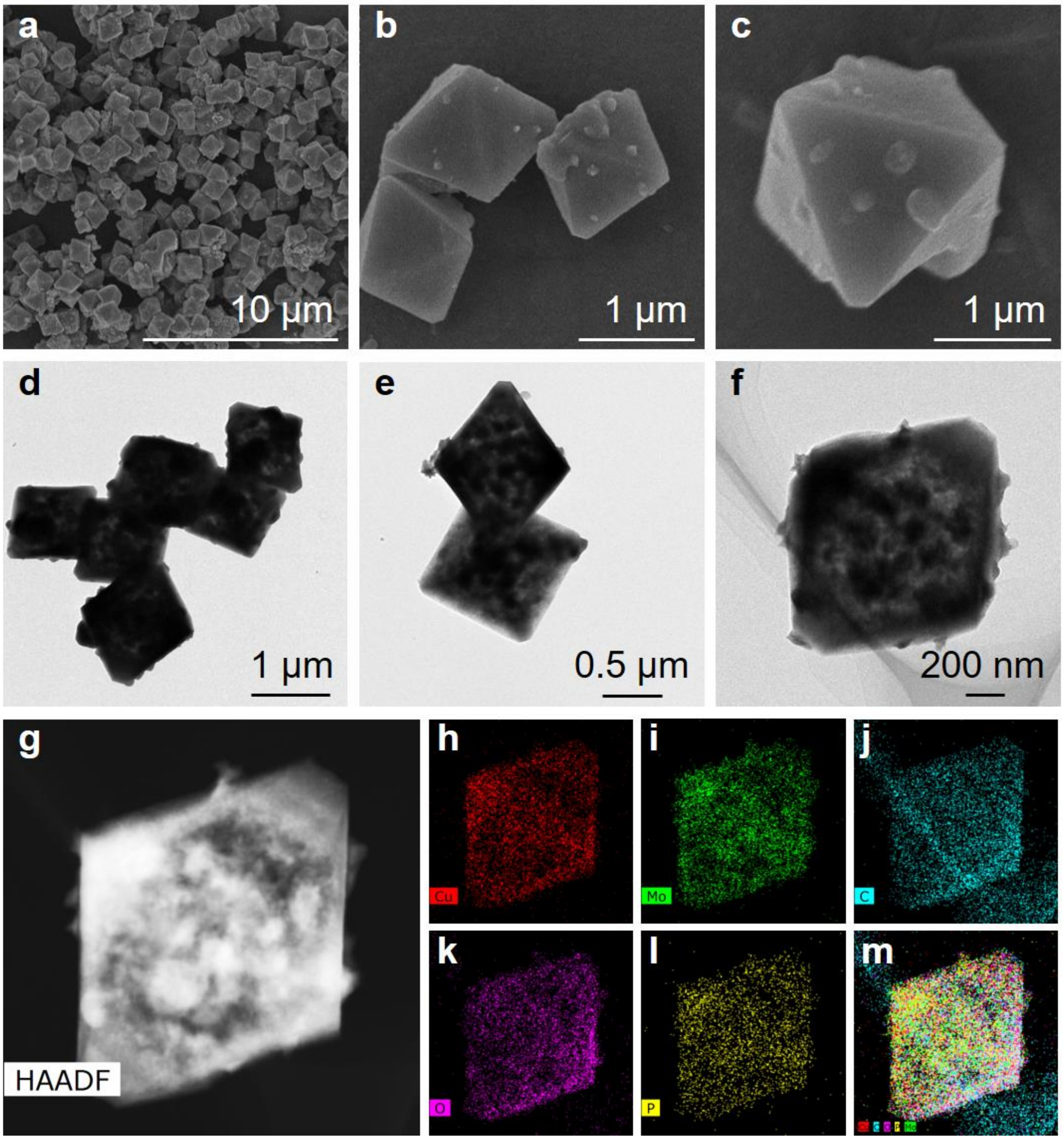

Supplementary Figure 7. SEM and TEM images of $Cu_x/MoO_2@C$. (a-c) SEM, (d-f) TEM, (g) HAADF-STEM images, and (h-m) the corresponding EDX mapping of $Cu_x/MoO_2@C$.

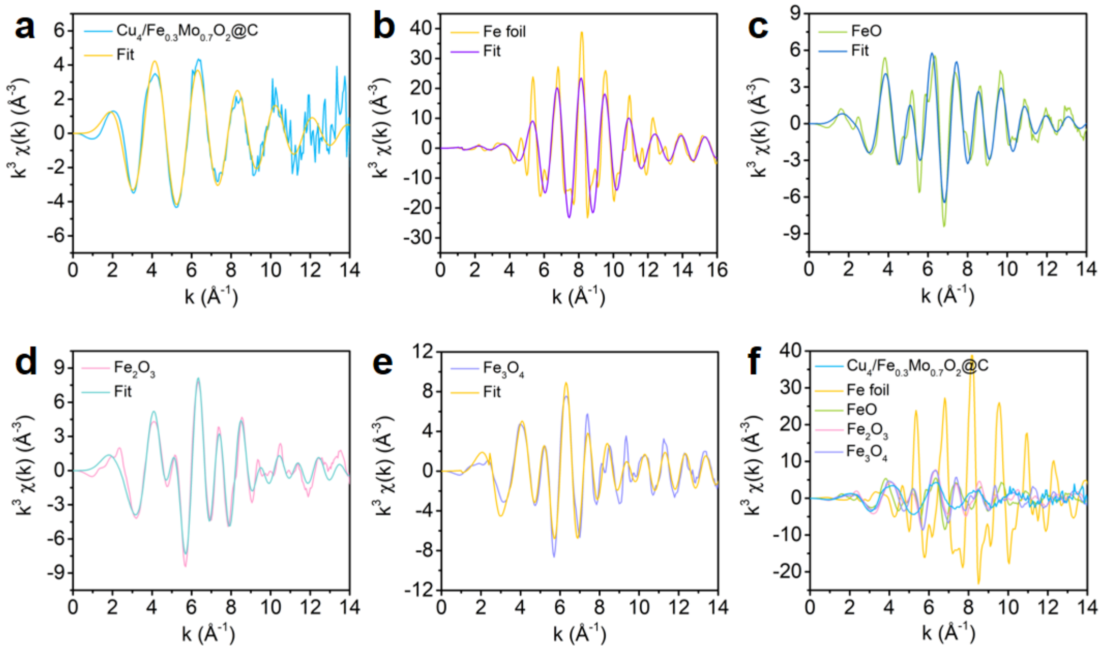

Supplementary Figure 8. XANES characterizations of $Cu_4/Fe_{0.3}Mo_{0.7}O_2@C$ and related counterparts. Fe K-edge EXAFS fitting curves of (a) $Cu_4/Fe_{0.3}Mo_{0.7}O_2@C$, (b) Fe foil, (c) FeO, (d) Fe_2O_3, and (e) Fe_3O_4. (f) K-space EXAFS curves at Fe foil, FeO, Fe_2O_3, Fe_3O_4, and $Cu_4/Fe_{0.3}Mo_{0.7}O_2@C$.

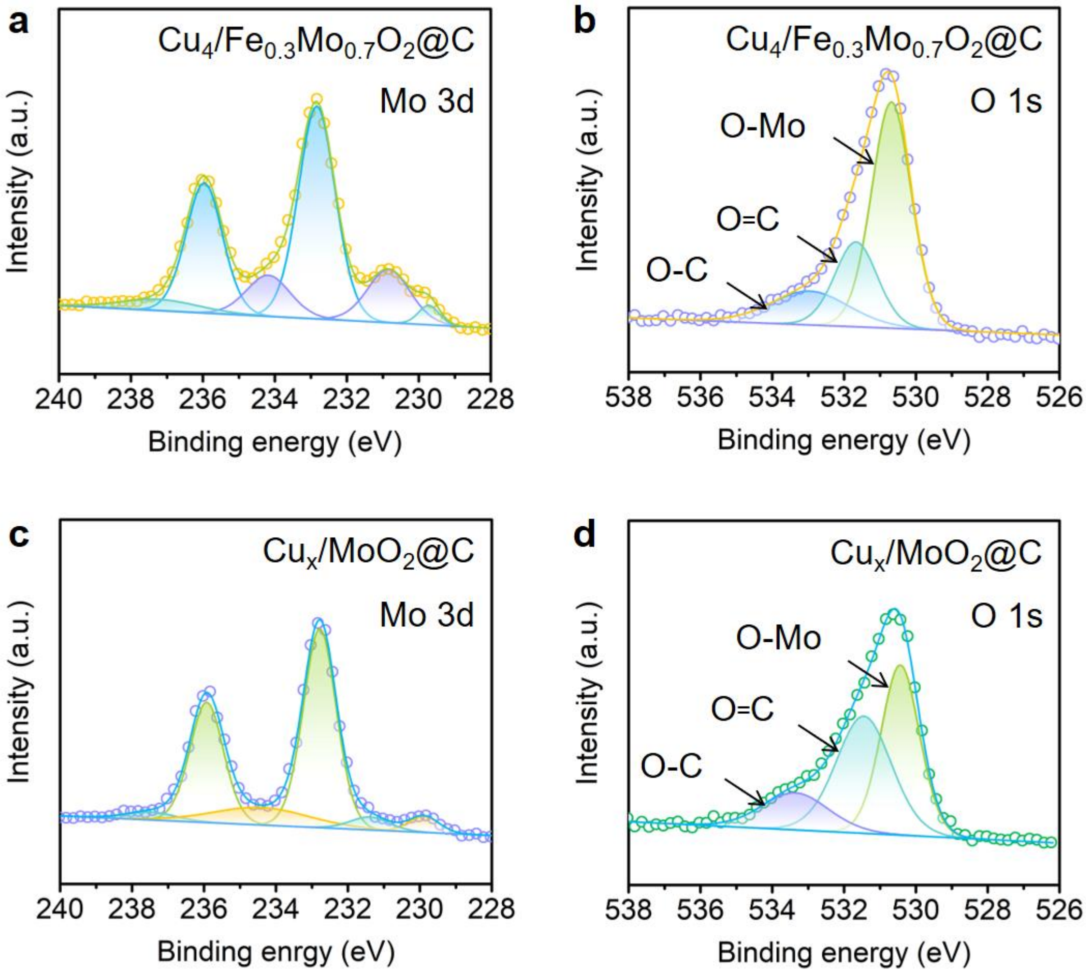

Supplementary Figure 9. XPS results of $Cu_4/Fe_{0.3}Mo_{0.7}O_2$@C and Cu_x/MoO_2@C. XPS spectra in Mo 3d region of (a) $Cu_4/Fe_{0.3}Mo_{0.7}O_2$@C and (c) Cu_x/MoO_2@C. XPS spectra in O 1s region of (b) $Cu_4/Fe_{0.3}Mo_{0.7}O_2$@C and (d) Cu_x/MoO_2@C.

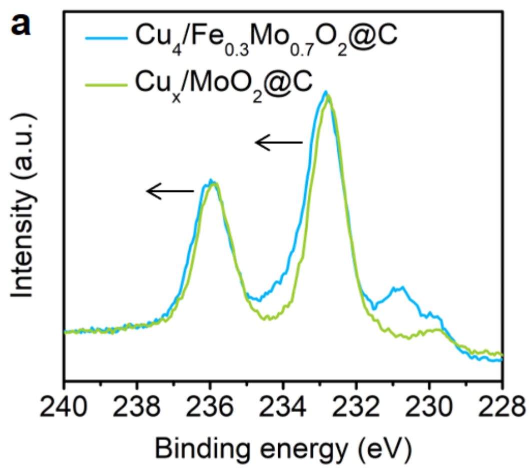

Supplementary Figure 10. XPS results of $Cu_4/Fe_{0.3}Mo_{0.7}O_2$@C and Cu_x/MoO_2@C. (a) Mo 3d and (b) O 1s XPS spectra for $Cu_4/Fe_{0.3}Mo_{0.7}O_2$@C (blue) and Cu_x/MoO_2@C (green).

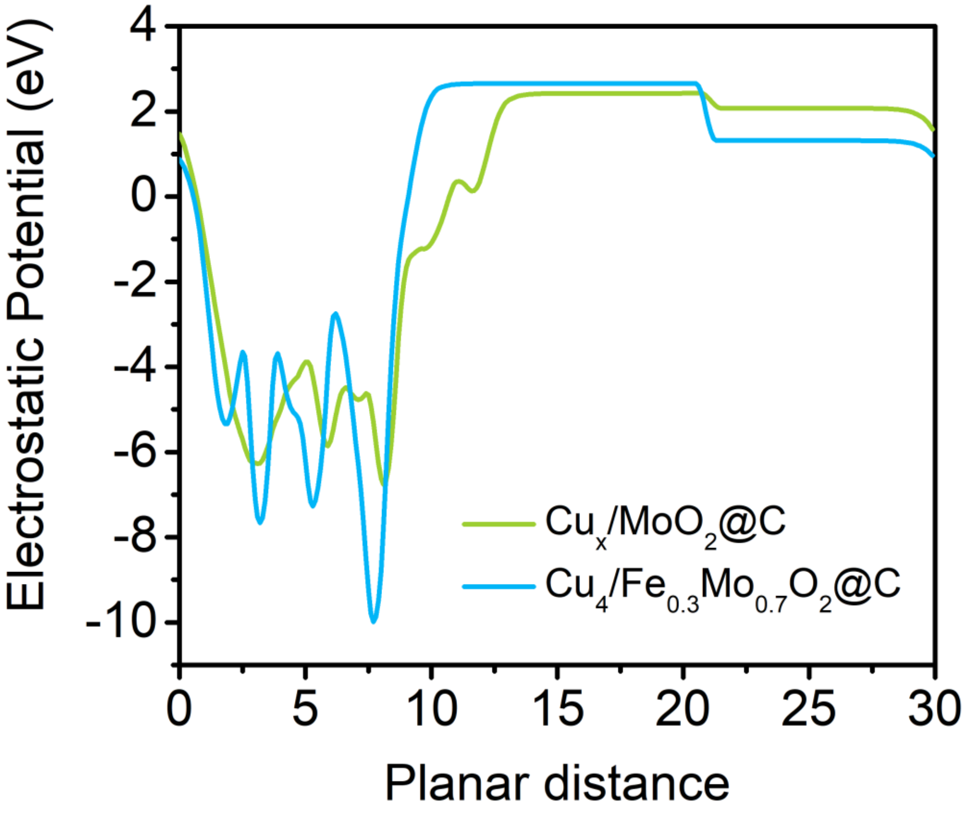

Supplementary Figure 11. The electrostatic potential at the surface of the $Cu_4/Fe_{0.3}Mo_{0.7}O_2$@C (blue) and Cu_x/MoO_2@C (green) models.

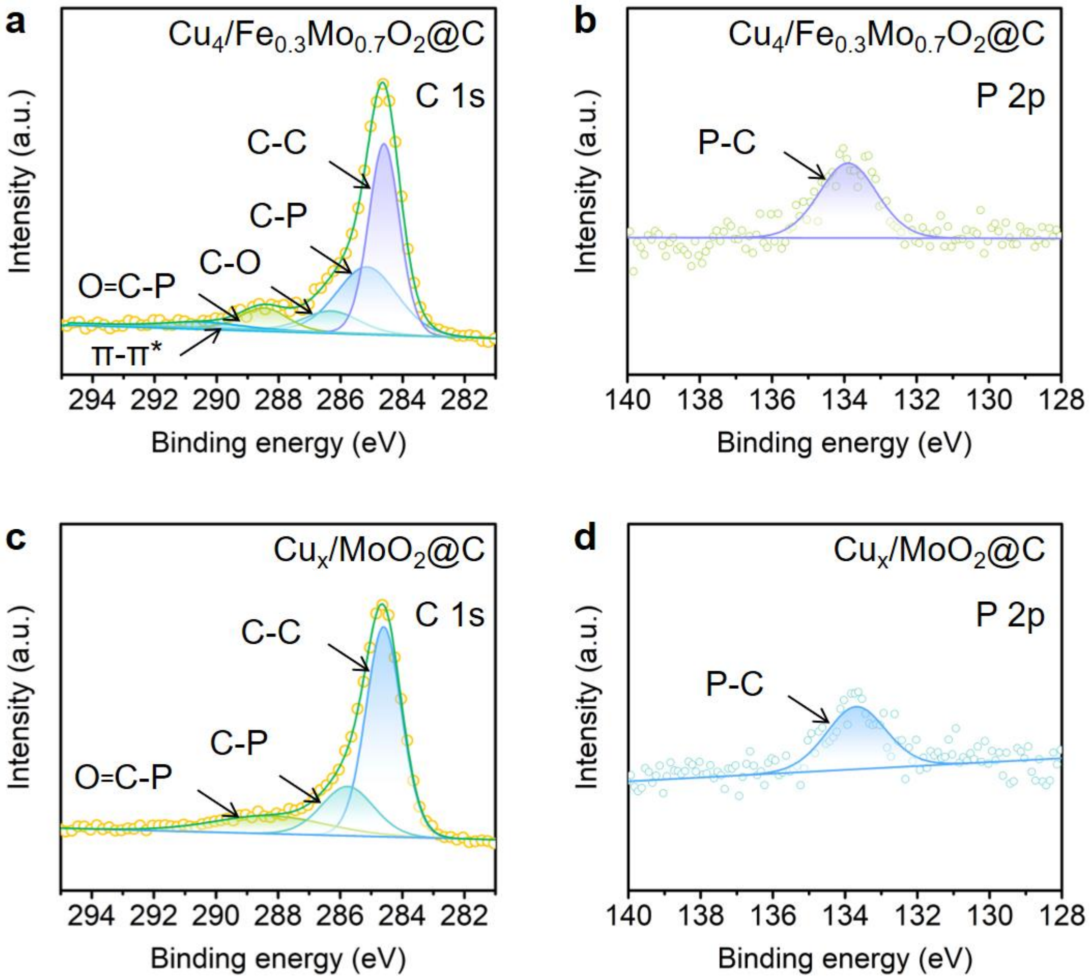

Supplementary Figure 12. XPS results of $Cu_4/Fe_{0.3}Mo_{0.7}O_2$@C and Cu_x/MoO_2@C. XPS spectra in C 1s region of (a) $Cu_4/Fe_{0.3}Mo_{0.7}O_2$@C and (c) Cu_x/MoO_2@C. XPS spectra in P 2p region of (b) $Cu_4/Fe_{0.3}Mo_{0.7}O_2$@C and (d) Cu_x/MoO_2@C.

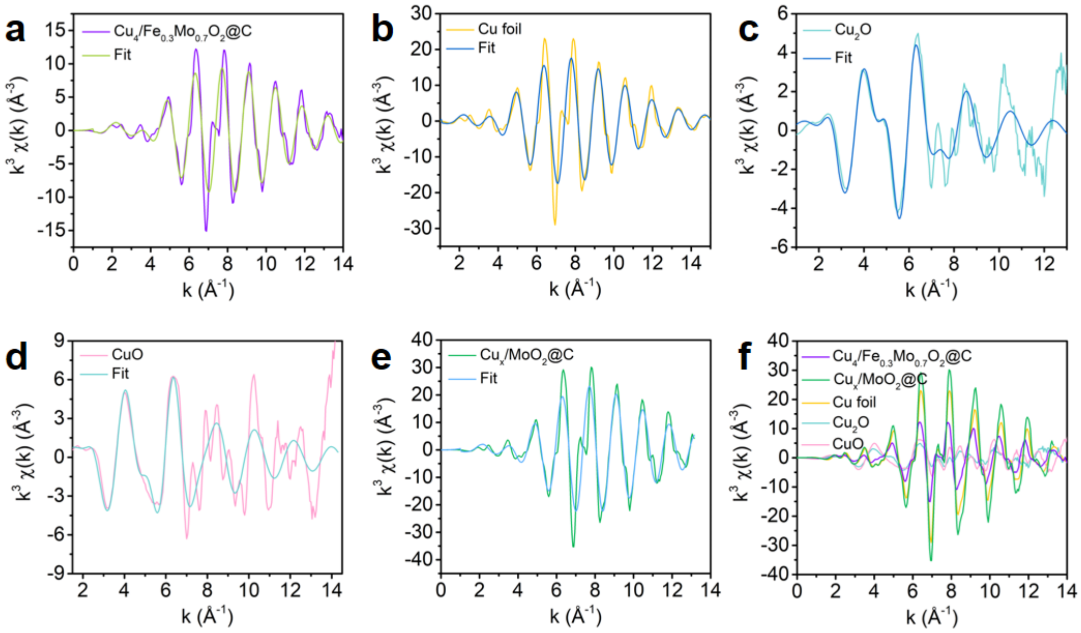

Supplementary Figure 13. XANES characterizations of $Cu_4/Fe_{0.3}Mo_{0.7}O_2@C$ and related counterparts. Cu K-edge EXAFS fitting curves of (a) $Cu_4/Fe_{0.3}Mo_{0.7}O_2@C$, (b) Cu foil, (c) Cu_2O, (d) CuO, and (e) $Cu_x/MoO_2@C$. (f) K-space EXAFS curves at Cu foil, Cu_2O, CuO, $Cu_x/MoO_2@C$, and $Cu_4/Fe_{0.3}Mo_{0.7}O_2@C$.

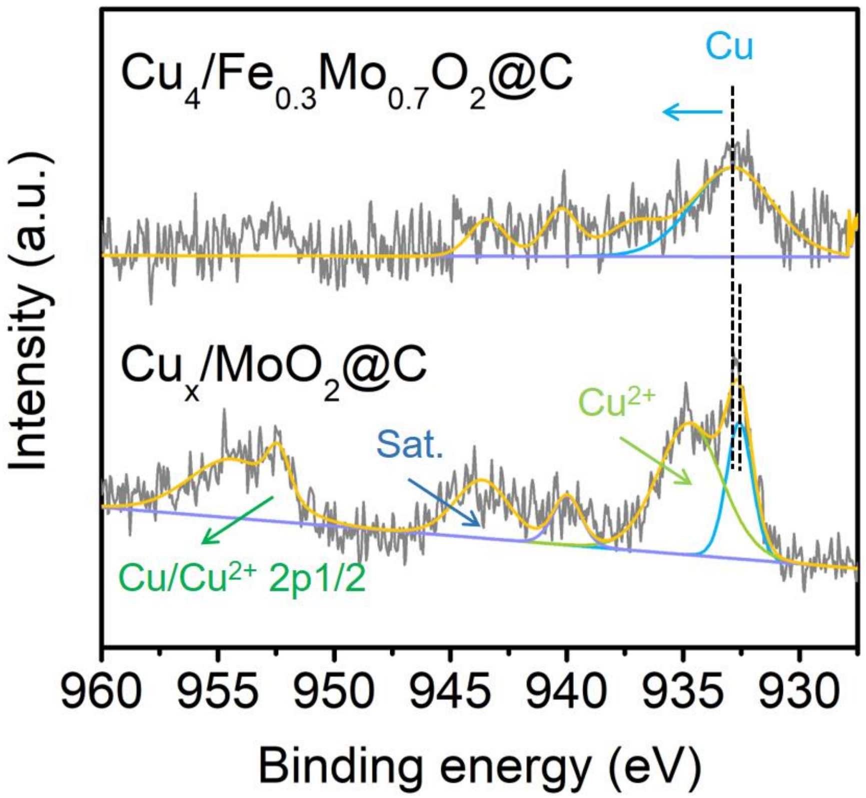

Supplementary Figure 14. XPS spectra in Cu 2p region of $Cu_4/Fe_{0.3}Mo_{0.7}O_2$@C and Cu_x/MoO_2@C.

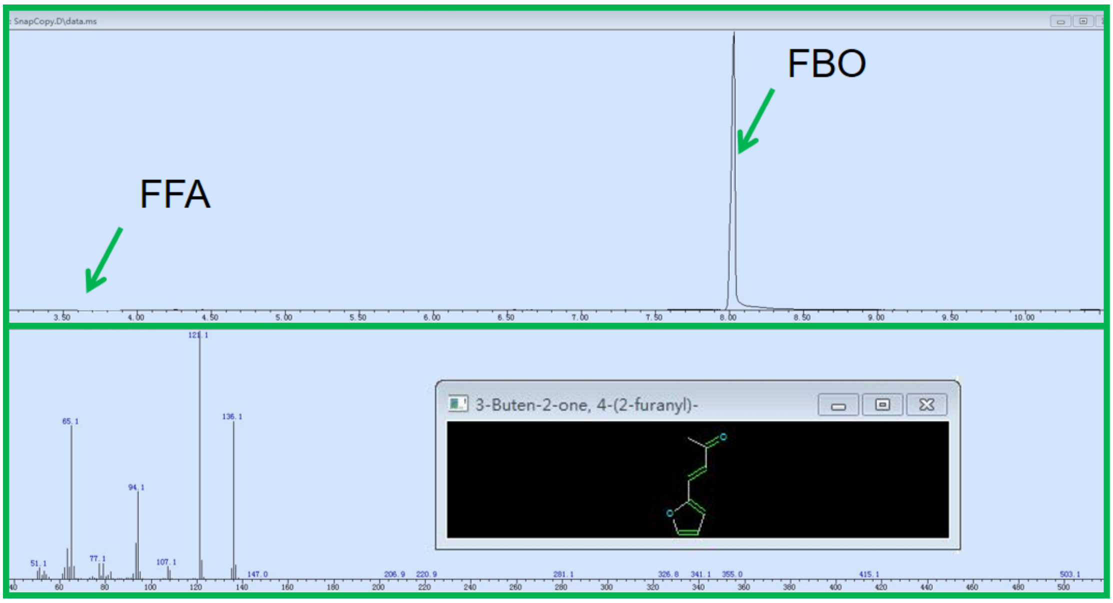

Supplementary Figure 15. A representative GC-MS spectrum for the determination of products (FBO) from the one-pot cascade oxidative coupling of FFA with IPA.

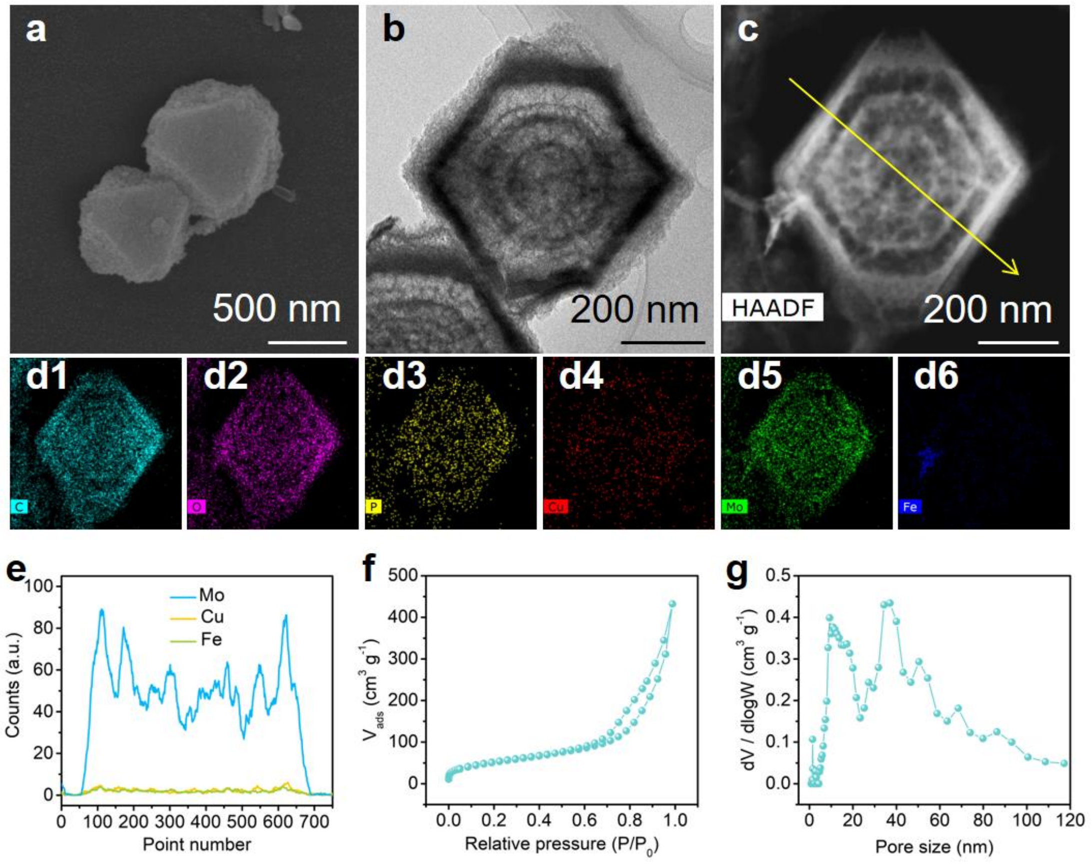

Supplementary Figure 16. Morphology and porosity characterizations of H-$Cu_4/Fe_{0.3}Mo_{0.7}O_2$@C. (a) SEM, (b) TEM, (c) HAADF-STEM, and (d1-d6) the corresponding elemental mapping images, (e) elemental line scan profiles, (f) N_2 adsorption-desorption isotherms, and (g) pore-size distributions of the as-synthesized H-$Cu_4/Fe_{0.3}Mo_{0.7}O_2$@C.

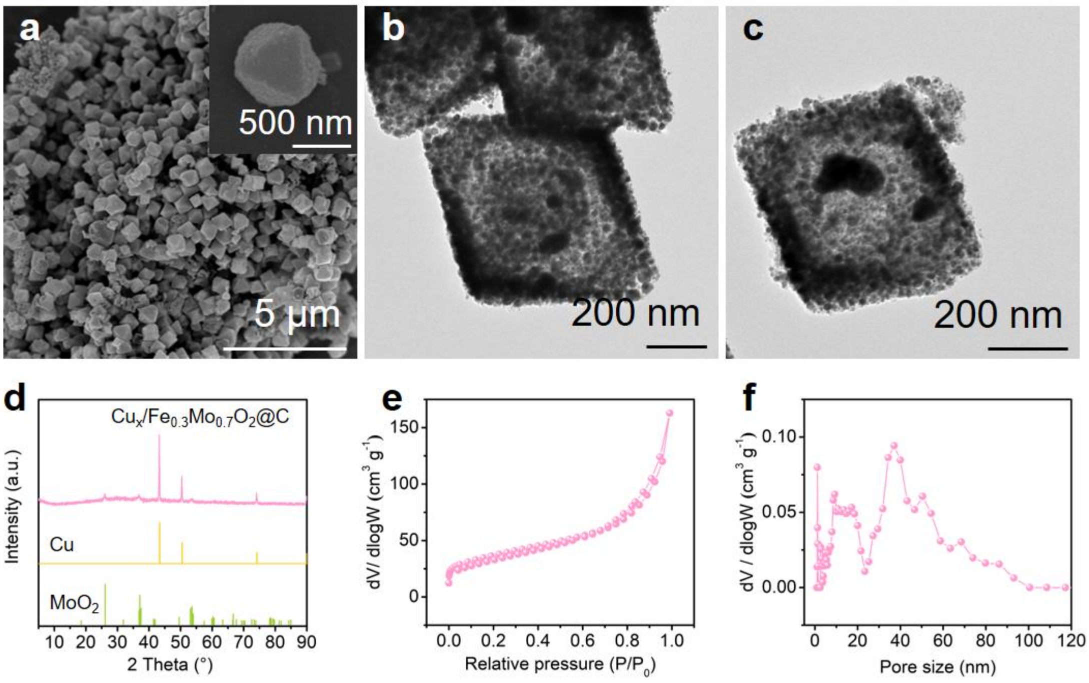

Supplementary Figure 17. Morphology and porosity characterizations of H-$Cu_x/Fe_{0.3}Mo_{0.7}O_2$@C. (a) SEM, (b, c) TEM images, (d) XRD patterns, (e) N_2 adsorption-desorption isotherms, and (f) pore-size distributions of the as-synthesized $Cu_x/Fe_{0.3}Mo_{0.7}O_2$@C.

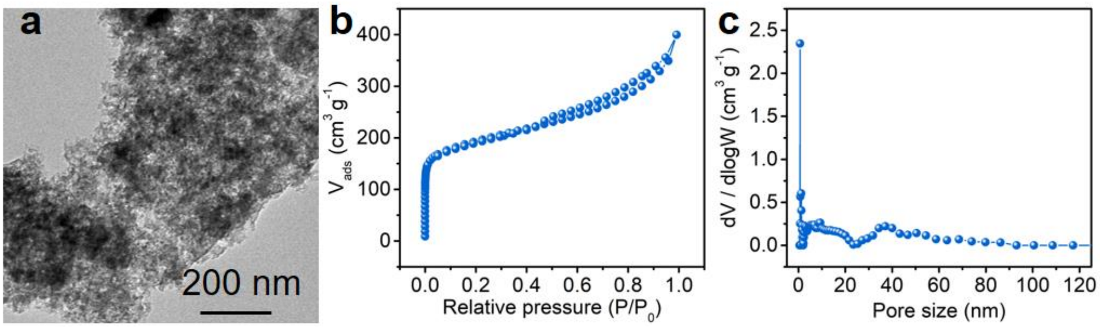

Supplementary Figure 18. Morphology and porosity characterizations of Cu-Fe-MoO_2. (a) TEM image, (b) N_2 adsorption-desorption isotherms, and (c) pore-size distributions of the Cu-Fe-MoO_2 (impregnation).

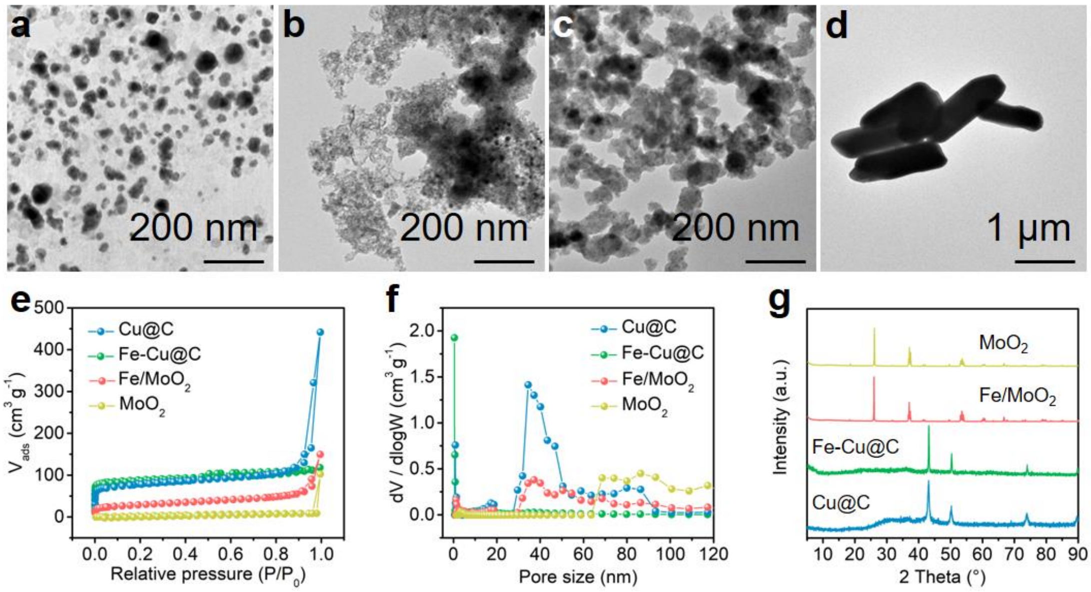

Supplementary Figure 19. Morphology and porosity characterizations of some counterparts. TEM images of (a) Cu@C, (b) Fe-Cu@C, (c) Fe/MoO_2, and (d) MoO_2. (e) N_2 adsorption-desorption isotherms, (f) pore-size distributions, and (g) XRD patterns of Cu@C, Fe-Cu@C, Fe/MoO_2, and MoO_2.

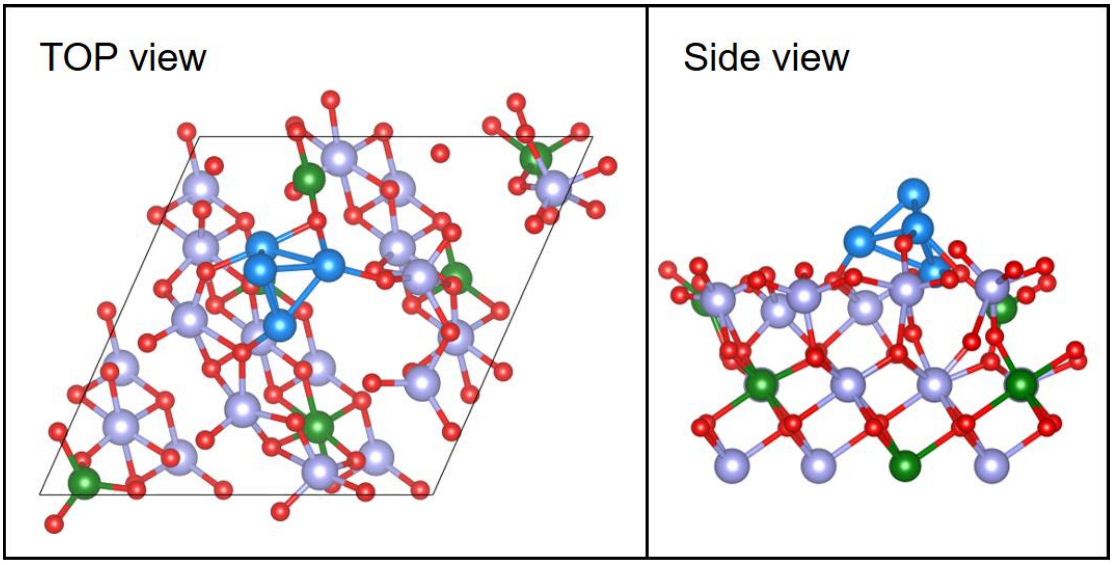

Supplementary Figure 20. The possible chemical structure of $Cu_4/Fe_{0.3}Mo_{0.7}O_2$@C (the structure model simplified as $Cu_4/Fe_{0.3}Mo_{0.7}O_2$). Blue, green, purple, and red balls represent Cu, Fe, Mo, and O atoms, respectively.

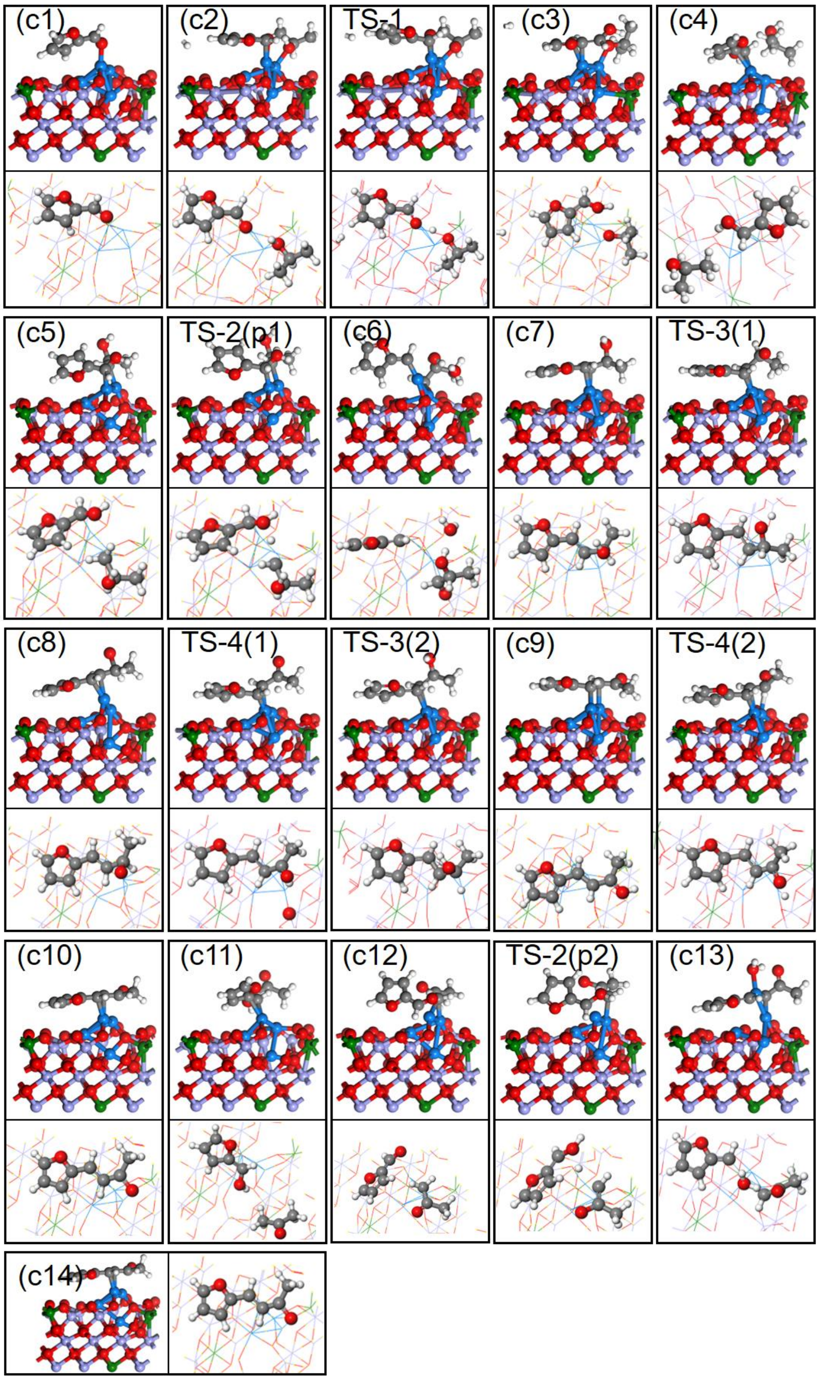

Supplementary Figure 21. Atomistic structures for one-pot cascade oxidative coupling of FFA with IPA into FBO over the $Cu_4/Fe_{0.3}Mo_{0.7}O_2@C$. (c1-c14) the simulated surface structures of various reaction species along the reaction pathway on $Cu_4/Fe_{0.3}Mo_{0.7}O_2@C$. The attached simplified structures are aiming to show the molecular formula of the reactants clearly. The blue, green, purple, red, gray, and white balls represent Cu, Fe, Mo, O, C, and H atoms, respectively. "TS" denotes a transition state.

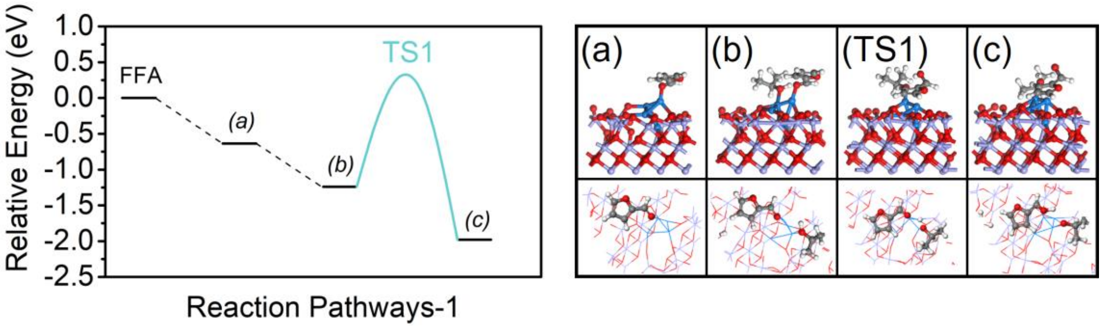

Supplementary Figure 22. The plausible reaction mechanism over Cu_4/MoO_2@C. The free energy diagram for oxidative coupling of FFA with IPA over Cu_4/MoO_2@C (left), the simulated and simplified structures of related states (a-c of right). The blue, purple, red, gray, and white balls represent Cu, Mo, O, C, and H atoms, respectively. "TS" denotes a transition state.

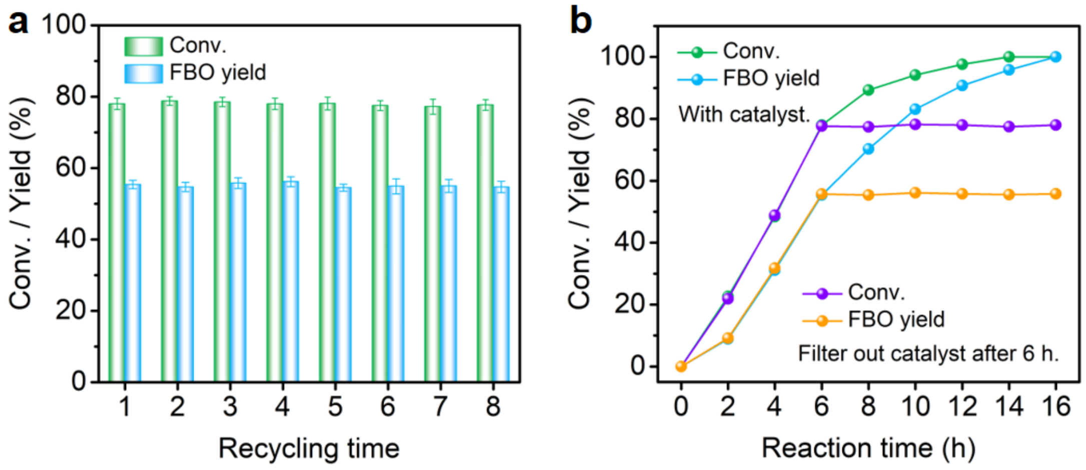

Supplementary Figure 23. Reusability examinations of the $Cu_4/Fe_{0.3}Mo_{0.7}O_2$@C. (a) Reusability results of the $Cu_4/Fe_{0.3}Mo_{0.7}O_2$@C catalyst in the oxidative coupling of FFA with IPA. Reaction conditions: furfural (0.5 mmol), catalyst (Cu, 3.6 mol%), K_2CO_3 (0.1 mmol), IPA (5 mL), O_2 (2 bar), 120 °C, 6 h. (b) The hot filtration experiment results for the oxidative coupling of FFA with IPA over $Cu_4/Fe_{0.3}Mo_{0.7}O_2$@C. Reaction conditions: furfural (0.5 mmol), catalyst (Cu, 3.6 mol%), K_2CO_3 (0.1 mmol), IPA (5 mL), O_2 (2 bar), 120 °C. Conversions and yields were determined by GC-MS. The error bars represent standard deviation based on three measurements.

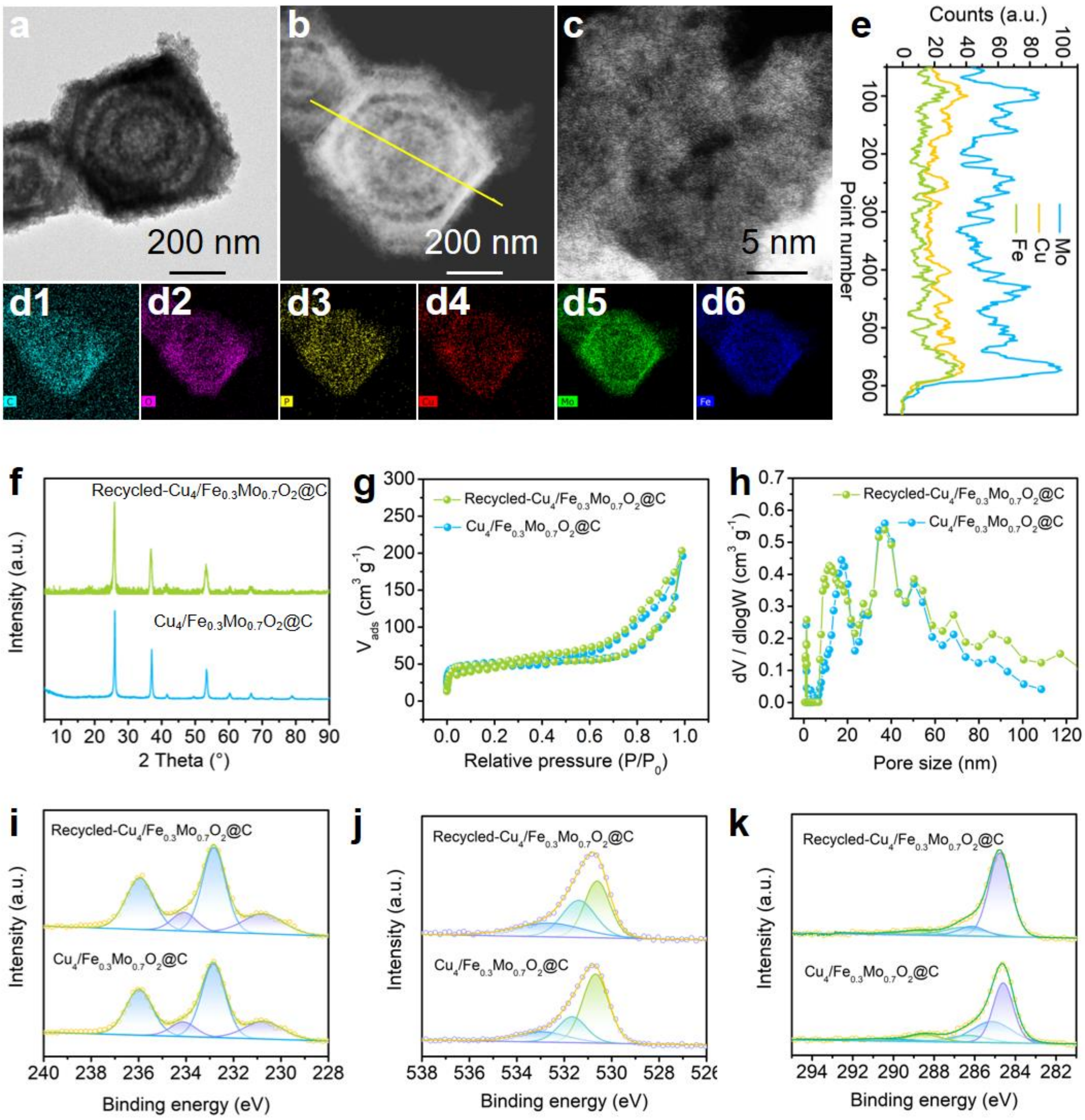

Supplementary Figure 24. Characterizations of the recycled $Cu_4/Fe_{0.3}Mo_{0.7}O_2$@C. (a) TEM, (b) HAADF-STEM, (c) AC HAADF-STEM, and (d) the corresponding EDX mapping images, (e) elemental line scan profiles, (f) XRD patterns, (g) N_2 adsorption-desorption isotherms, (h) pore-size distributions, XPS spectra of (i) Mo 3d, (j) O 1s, and (k) C 1s regions of the fresh and recycled $Cu_4/Fe_{0.3}Mo_{0.7}O_2$@C SHHPO.

Supplementary Table 1. Characterization results of the as-prepared samples.

Sample	S_{BET} (m^2g^{-1})	Pore volume (cm^3g^{-1})	Element contents (wt%)					
			Cu	Mo	Fe	C	O	P
NENU-5	615.2	0.34	–	–	–	–	–	–
Fe-NENU-5	399.3	0.39	–	–	–	–	–	–
Cu_x/MoO_2@C	182.9	0.24	7.2	16.4	–	50.3	23.4	2.4
$Cu_4/Fe_{0.3}Mo_{0.7}O_2$@C	169.7	0.41	4.8	15.6	3.6	51.8	20.1	2.7
$Cu_x/Fe_{0.3}Mo_{0.7}O_2$@C	127.2	0.17	8.0	15.3	3.2	49.4	20.2	2.3
H-$Cu_4/Fe_{0.3}Mo_{0.7}O_2$@C	210.9	0.44	–	16.2	3.9	52.2	24.0	2.3
Cu-Fe-MoO_2 (impregnation)	627.7	0.16	4.5	–	3.5	–	–	–
Cu@C	256.0	0.22	54.7	–	–	41.4	–	–
Fe-Cu@C	274.8	0.18	14.8	–	30.6	50.5	–	–
Fe/MoO_2	101.2	0.13	–	–	3.9	–	–	–
MoO_2	17.7	0.01	–	–	–	–	–	–
Recycled-$Cu_4/Fe_{0.3}Mo_{0.7}O_2$@C	163.0	0.40	4.8	15.8	3.5	51.1	20.6	2.5

Supplementary Table 2. Structural parameters of various samples extracted from the EXAFS fitting (S_0^2=0.80).

Sample	Path	C.N.	R (Å)	$\sigma^2 \times 10^3$ (Å^2)	ΔE (eV)	R factor
Fe foil	Fe-Fe	8*	2.47±0.01	5.0±0.4	6.1±0.8	0.002
	Fe-Fe	6*	2.84±0.01	6.2±0.8	4.5±1.7	
FeO	Fe-O	4.8±1.2	2.11±0.02	10.1±2.8	0.8±3.0	0.010
	Fe-Fe	10.5±1.4	3.07±0.01	11.1±1.1	1.5±1.3	
Fe_2O_3	Fe-O	6.2±1.3	1.93±0.02	11.8±2.4	-5.7±3.0	0.015
	Fe-Fe	2.6±2.4	2.99±0.05	8.8±5.0	-4.2±8.2	
	Fe-Fe	8.5±4.5	3.42±0.02	10.3±.2	-7.9±3.3	
Fe_3O_4	Fe-O	6.8±2.1	2.04±0.03	17.4±4.5	4.0±3.6	0.016
	Fe-Fe	5.2±2.7	3.24±0.04	5.4±4.2	10.9±2.3	
	Fe-Fe	6.9±4.2	3.38±0.07	5.1±2.5		
$Cu_4/Fe_{0.3}Mo_{0.7}O_2$@C	Fe-O	4.9±0.3	1.98±0.01	6.5±0.6	-3.6±0.8	0.005

[a]*C.N.*: coordination numbers; [b]*R*: bond distance; [c]σ^2: Debye-Waller factors; [d]ΔE: the inner potential correction. *R* factor: goodness of fit. *The experimental EXAFS fit of metal foil by fixing CN as the known crystallographic value.

Supplementary Table 3. Structural parameters of various samples extracted from the EXAFS fitting (S_0^2=0.91).

Sample	Path	C.N.	R (Å)	$\sigma^2 \times 10^3$ (Å^2)	ΔE (eV)	R factor
Cu foil	Cu-Cu	12*	2.52±0.01	8.4±0.3	4.4±0.5	0.002
Cu_2O	Cu-O	3.2±0.4	1.92±0.01	7.2±1.3	0.2±1.3	0.009
	Cu-Cu	11.5±3.2	3.02±0.02	32.7±3.6	2.2±1.4	
CuO	Cu-O	4.1±0.8	1.95±0.01	5.1±2.1	1.0±2.0	0.019
	Cu-Cu	12.1±2.6	2.97±0.04	33.2±8.4	0.0±3.4	
$Cu_x/MoO_2@C$	Cu-Cu	12.5±0.7	2.54±0.01	7.6±0.3	3.9±0.5	0.002
$Cu_4/Fe_{0.3}Mo_{0.7}O_2@C$	Cu-O	0.5±0.3	1.90±0.04	3.6±4.9	6.3±6.7	0.003
	Cu-Cu	6.3±0.4	2.54±0.01	7.7±0.4	4.2±0.8	

[a]*C.N.*: coordination numbers; [b]*R*: bond distance; [c]σ^2: Debye-Waller factors; [d]ΔE: the inner potential correction. *R* factor: goodness of fit. *The experimental EXAFS fit of metal foil by fixing CN as the known crystallographic value.

Supplementary Table 4. Reaction results of the oxidative coupling of FFA with IPA into FBO over different catalysts.

Entry	Catalysts	Conv. (%)	Yield (%)					
			FFAL	FBOL	**FBO (Sel.)**	IFC	DPMF	FYBO
1	–	–	–	–	–	–	–	–
2	$Cu_4/Fe_{0.3}Mo_{0.7}O_2$@C	100	–	–	>99 (>99)	–	–	–
3	Cu_x/MoO_2@C	59.8	4.3	–	41.3 (69.1)	7.4	–	6.8
4	H-$Cu_4/Fe_{0.3}Mo_{0.7}O_2$@C	30.0	–	15.6	6.3 (21.0)	–	8.1	–
5	$Cu_x/Fe_{0.3}Mo_{0.7}O_2$@C	71.9	3.4	–	55.5 (77.2)	5.1	–	7.9
6	Cu@C	60.3	2.2	–	34.9 (57.9)	23.2	–	–
7	Fe-Cu@C	38.2	5.8	–	21.9 (57.3)	7.2	3.3	–
8	Cu-Fe-MoO_2	33.9	5.9	1.4	20.6 (60.8)	3.9	2.1	–
9	Fe/MoO_2	42.4	1.7	19.5	10.9 (25.7)	–	8.7	1.6
10	MoO_2	24.8	–	13.2	4.7 (18.9)	–	6.9	–

Reaction conditions: furfural (0.5 mmol), catalysts (Cu, 3.6 mol%), K_2CO_3 (0.1 mmol), IPA (5 mL), O_2 (2 bar), 120 °C, 16 h. Conversion and yield were determined by GC-MS. For the control catalysts without Cu species, the catalyst usage 0.03 g.

Supplementary Table 5. Reaction results of the synthesis of FBO using different substrates over $Cu_4/Fe_{0.3}Mo_{0.7}O_2@C$. [a]

Entry	Substrate		Conv. (%)	Yield (%)
1[b]	FFA	acetone	15.8	14.7
2	FFAL	acetone	2.0	2.0
3	FFAL	IPA	31.9	31.9
4[c]	FFA	IPA	5.0	5.0

[a]Reaction conditions: FFA or FFAL (0.5 mmol), catalysts (Cu, 3.6 mol%), K_2CO_3 (0.1 mmol), IPA or acetone (5 mL), O_2 (2 bar), 120 °C, 16 h. [b] FFA (0.25 mmol), acetone (0.25 mmol). [c]N_2 (2 bar). Conversion and yield were determined by GC-MS.

Supplementary Table 6. One-pot cascade oxidative coupling of FFA with various secondary alcohols over $Cu_4/Fe_{0.3}Mo_{0.7}O_2@C$. [a]

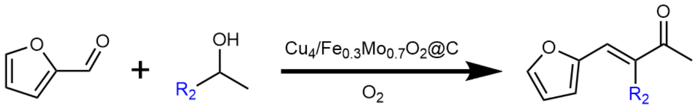

1a (2-Butanol)

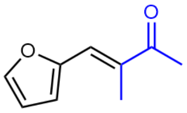

Conv. = 100%

Yield = 97.5%

2a (2-Pentanol) [b]

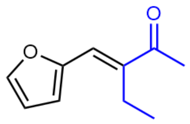

Conv. = 100%

Yield = 94.8%

3a (2-Hexanol) [c]

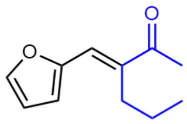

Conv. = 97.4%

Yield = 95.1%

4a (2-Heptanol) [d]

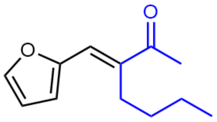

Conv. = 97.7%

Yield = 91.2%

5a (2-Octanol) [d]

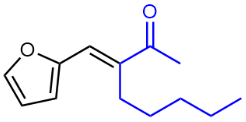

Conv. = 95.1%

Yield = 86.7%

6a (2-Nonanol) [e]

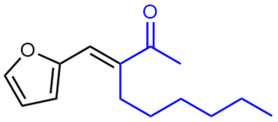

Conv. = 89.4%

Yield = 83.3%

7a (2-Decanol) [e]

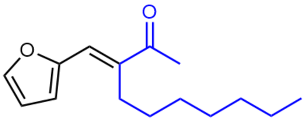

Conv. = 84.6%

Yield = 81.7%

[a]Reaction conditions: furfural (0.5 mmol), catalyst (Cu, 3.6 mol%), K_2CO_3 (0.1 mmol), alcohol (5 mL), O_2 (2 bar), 120 °C, 16 h. Conversions and yields were determined by GC-MS. [b]130 °C, 24 h. [c]130 °C, 36 h. [d]150 °C, 36 h. [e]150 °C, 48 h.

Supplementary Table 7. One-pot cascade oxidative coupling of FFA with various primary alcohols over $Cu_4/Fe_{0.3}Mo_{0.7}O_2@C$. [a]

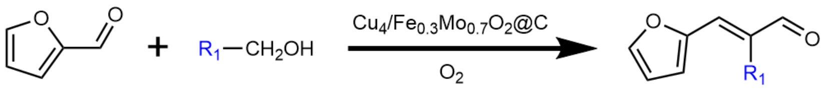

8b (1-Propanol)

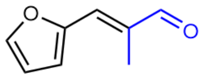

Conv. = 100%

Yield > 99%

9b (1-Butanol) [b]

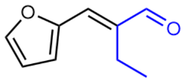

Conv. = 100%

Yield = 98.2%

10b (1-Pentanol) [b]

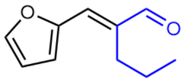

Conv. = 100%

Yield = 95.5%

11b (1-Hexanol) [b]

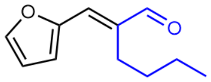

Conv. = 100%

Yield = 92.3%

12b (1-Heptanol) [c]

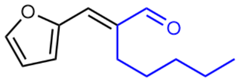

Conv. = 100%

Yield = 93.1%

13b (1-Octanol) [c]

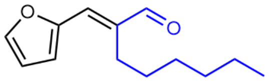

Conv. = 96.4%

Yield = 90.9%

14b (1-Nonanol) [d]

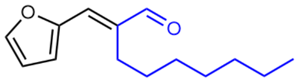

Conv. = 97.8%

Yield = 88.9%

15b (1-Decanol) [d]

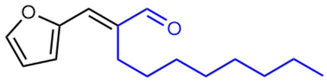

Conv. = 96.9%

Yield = 82.8%

[a]Reaction conditions: furfural (0.5 mmol), catalyst (Cu, 3.6 mol%), K_2CO_3 (0.1 mmol), alcohol (5 mL), O_2 (2 bar), 120 °C, 16 h. Conversions and yields were determined by GC-MS. [b]120 °C, 24 h. [c]140 °C, 24 h. [d]150 °C, 36 h.

Supplementary Table 8. Raw data of Bader charge analysis.

	X	Y	Z	Charge
1	3.3471	2.6843	16.3719	6.7971
2	2.2259	3.3256	19.0309	6.6542
3	2.9429	-0.9553	21.4124	6.7373
4	0.4097	5.9307	21.5434	6.8125
5	4.5484	0.0473	16.5582	6.7284
6	5.0416	1.8211	19.2843	6.8774
7	0.2125	3.2444	21.4124	6.8519
8	3.8901	6.117	15.7183	6.7871
9	5.0294	-0.9325	19.1429	6.9548
10	1.5759	4.9445	16.688	6.8409
11	2.4014	0.4023	19.3369	6.851
12	3.14	1.731	21.5434	6.9105
13	7.1152	-1.2647	16.8375	6.6548
14	7.5308	0.8073	18.9951	6.9833
15	8.4036	-3.3971	21.4124	6.9421
16	5.8704	3.4889	21.5434	6.8294
17	0.4931	2.1036	16.7444	6.853
18	10.549	-0.6597	19.2901	6.7344
19	5.6733	0.8026	21.4124	6.8702
20	9.5243	0.7981	16.6295	6.6215
21	10.3209	-3.5044	19.2308	6.8554
22	6.6971	1.5883	16.7336	6.8839
23	7.8204	-2.2689	19.2977	6.9184
24	8.6008	-0.7109	21.5434	6.9148
25	0.1967	8.6839	15.8358	6.6116
26	2.4517	9.8083	18.8472	6.8415
27	2.9429	5.0023	21.4124	6.9516
28	0.4097	-0.0269	21.5434	6.9213
29	2.1077	7.838	17.2619	6.8197
30	4.9965	7.9276	19.454	6.8385
31	0.2125	9.202	21.4124	6.8218
32	4.7026	8.9974	16.8505	6.5914
33	4.2364	5.6409	18.4546	6.8542
34	2.3216	10.5965	16.3829	6.8393
35	1.961	6.1673	19.3312	6.8978
36	3.14	7.6886	21.5434	6.9014
37	6.6397	6.6323	16.5661	6.8642
38	7.7312	6.8051	19.2517	6.8309
39	8.4036	2.5604	21.4124	6.9324
40	5.8704	-2.4687	21.5434	6.9237

41	8.5673	3.4837	16.514	6.8959
42	10.8682	4.8649	18.971	6.973
43	5.6733	6.7601	21.4124	6.9261
44	10.6329	6.2383	16.8093	6.8753
45	9.6605	2.6003	18.9226	6.8477
46	8.7818	-3.1497	16.5163	6.7835
47	7.3238	3.656	19.269	6.8304
48	8.6008	5.2467	21.5434	6.9208
49	5.1824	2.6565	16.1454	10.7962
50	5.4478	5.0468	15.5107	10.8024
51	7.6341	5.0754	16.695	10.8148
52	7.1526	3.4394	15.0043	10.9018
53	4.0402	0.4472	20.1992	3.9209
54	10.087	-4.0832	17.4982	4.1389
55	1.3098	10.6045	20.1992	4.1043
56	2.0427	6.286	22.7569	5.0639
57	7.4735	7.847	17.6186	4.4115
58	7.5035	3.8442	22.7569	5.0567
59	3.9264	-1.5325	17.504	3.9765
60	4.7731	2.0863	22.7569	5.0268
61	1.8838	3.1652	17.23	3.8385
62	1.3098	4.647	20.1992	3.941
63	2.0427	0.3285	22.7569	5.053
64	9.5009	-1.9946	20.1992	3.9519
65	10.2338	-0.3555	22.7569	5.0396
66	6.15	-0.1199	17.6482	4.0154
67	6.7705	2.2051	20.1992	3.904
68	7.5035	-2.1134	22.7569	5.0355
69	2.8992	6.2475	17.1929	3.8332
70	4.0402	6.4048	20.1992	4.0549
71	4.7731	8.0439	22.7569	5.0397
72	0.8913	9.5737	17.1122	3.8356
73	8.529	1.8822	17.4866	3.8322
74	9.5009	3.963	20.1992	4.0943
75	10.2338	5.6021	22.7569	5.0695
76	6.7705	8.1627	20.1992	4.093
